# *Parthenium hysterophorus* steps up Ca-regulatory pathway in defence against highlight intensities

**DOI:** 10.1038/s41598-020-65721-7

**Published:** 2020-06-02

**Authors:** Javed Ahmad, M. Affan Baig, Amna, Ibrahim A. Alaraidh, Abdulaziz A. Alsahli, M. Irfan Qureshi

**Affiliations:** 1 Department of Biotechnology, Jamia Millia Islamia, New Delhi, New Delhi 110025 India; 2 Botany & Microbiology Department, Science College, King Saud University, P.O. Box, 2455 Riyadh, Saudi Arabia

**Keywords:** Light responses, Environmental sciences

## Abstract

*Parthenium hysterophorus* exhibits tolerance to a great extent against abiotic stresses including high light intensities. In this study, *P. hysterophorus* was subjected to three different light intensities viz. control (CL, 250 µmol photons m^−2^ s^−1^), moderately high (ML, 500 µmol photons m^−2^ s^−1^) and high (HL, 1000 µmol photons m^−2^ s^−1^) for assessment of biochemical and physiological responses at 3 and 5 days after treatment (DAT). Proteomic responses were also observed at 5 DAT. Level of oxidative stress marker, abundance of H_2_O_2_ and O_2_^−^ was highest in leaves exposed to HL followed by ML treatment. Biomass accumulation, photosynthetic parameters, chloroplast and mitochondrial integrity were also affected by both ML and HL treatments. Differential protein expression data showed modulation of thirty-eight proteins in ML and HL intensities. *P. hysterophorus* exhibited good ability to survive in ML then HL treatment as demonstrated by enhancement of the antioxidant system and photosynthesis. Furthermore, *P. hysterophorus* mobilized some key proteins related to calcium signaling, which in turn coordinate physiological homeostasis under stress. Proline and total soluble sugar content were high under stress; however, results of simulated experiment of our study indicate such accumulation of osmolytes may inhibit photon-availability to chloroplast. These results clarify our understanding of the mechanisms underlying the light stress tolerance of *P. hysterophorus*.

## Introduction

Light is one of the primary essential environmental factors that affect growth and development of plants. Any slight change in light intensity, duration and quality can influence the rate, pattern of growth and development which can significantly influence the productivity at mass scale^1,2^. Sensitivity of a plant to high intensities of light may lead to failure in acclimation responses leading to cellular damage and ultimately to the death of plants^3^. As the light energy absorption exceeds capacity of its usage in the chloroplasts, oxygenic photosynthesis electron transport is elevated, generating excessive reactive oxygen species (ROS) through photochemical energy conversion^4^. ROS damages cellular components including photosynthetic reaction centers and peripherical light-harvesting structures. Such light-induced damages might decrease photosynthetic activity, known as photoinhibition which is still poorly understood^5,6^. Also, how do plants respond to highlight intensities at a cellular level and what might be rescue mechanisms are still in need of full understanding. Under light stress, role of calcium could also be crucial in protection of plants and thylakoids^7,8^ besides other protective mechanisms^1,9–11^. Such mechanisms primarily, and effectively, include defence pathways those operate through modulation of proteome profiles^12–14^. The efficiency of defense depends on degree of modulation, and qualitative composition of a proteome decides the degree of threshold to withstand abiotic stress.

Compared to crop plants, weeds are generally much more tolerant to abiotic stresses^15^. Among such plants is an invasive weed *Parthenium hysterophorus* (Congress grass or Gajar Ghas), a member of the Asteraceae. It has special morpho-physiological and biochemical adaptabilities, stress tolerance and biosynthesizes novel secondary metabolites for its own defence^16–18^. All these special features contribute to *P. hysterophorus* growing well in varied habitats and harsh ecological conditions (e.g., high light intensities, drought, heat, etc.) enabling *P. hysterophorus* to become a global weed^15^.

Combination of physiological and proteomic analysis might provide valuable information about regulatoryprocesses, which are important for understanding stress physiology regulation and the performance of crops. A lot of work has been done on crop and horticulture plants, but weeds are widely neglected even when they are the most eligible candidates to study stress-tolerance mechanisms concerning a single or variety of stresses. Therefore, the present study focuses on working out the identity of key protein players and their contribution of high light stress response of*P. hysterophorus* during the high light stress. A comparative account of levels of oxidative stress, histochemical detection of ROS, osmolyte accumulation, calcium content, cellular antioxidant capacity, chlorophyll content, chloroplast and mitochondrial ultrastructure, photosynthesis related parameters and biomass accumulation data accompanying physiological responses to moderate and high intensities of lights has been analyzed for *P. hysterophorus*.

## Results

### TBARS as oxidative stress marker

**Figure 1:**
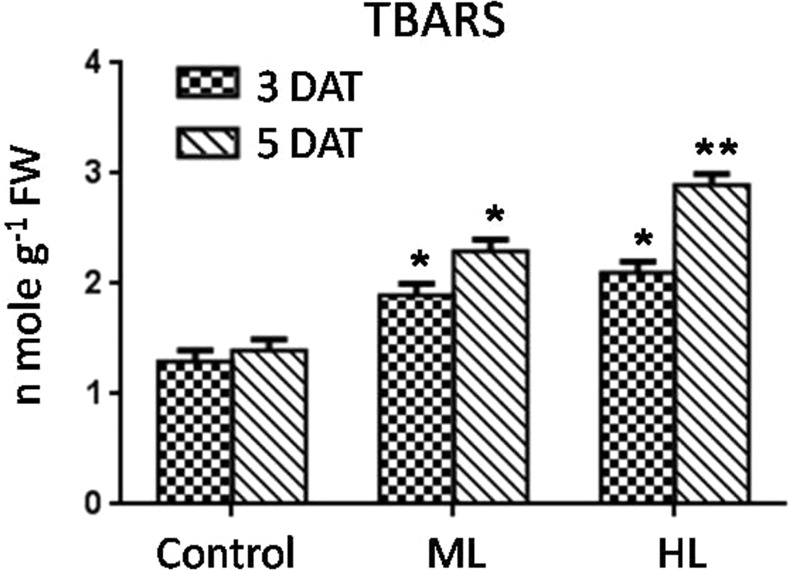
Effect of control (Control, 250 µmol photons m^−2^ s^−1^), moderate (ML, 500 µmol photons m^−2^ s^−1^) and high (HL, 1000 µmol photons m^−2^ s^−1^) light intensities on content of thiobarbituric acid substances (TBARS) showing the magnitude of oxidative damage in leaf of *Parthenium hysterophorus* at 3 DAT and 5 DAT. Values are mean ± SD and n = 5.

Magnitude of oxidative stress was measured in terms of stress-damaged byproduct’s reaction with thiobarbuteric acid (TBA) to form colored TBA-reactive substances (TBARS). Impact of normal, moderate light (ML) and high light (HL) intensities-induced oxidative stress in *P. hysterophorus* was assessed. As compared to control plants, TBARS increased 46% and 57% under ML and 62% and 93% under HL at 3 DAT and 5 DAT, respectively (Fig. 1). Such increasing concentration of TBARS in *P. hysterophorus* suggested ML and HL induced oxidative stress.

### HL stress induced higher histological accumulation of hydrogen peroxide (H_2_O_2_)

**Figure 2:**
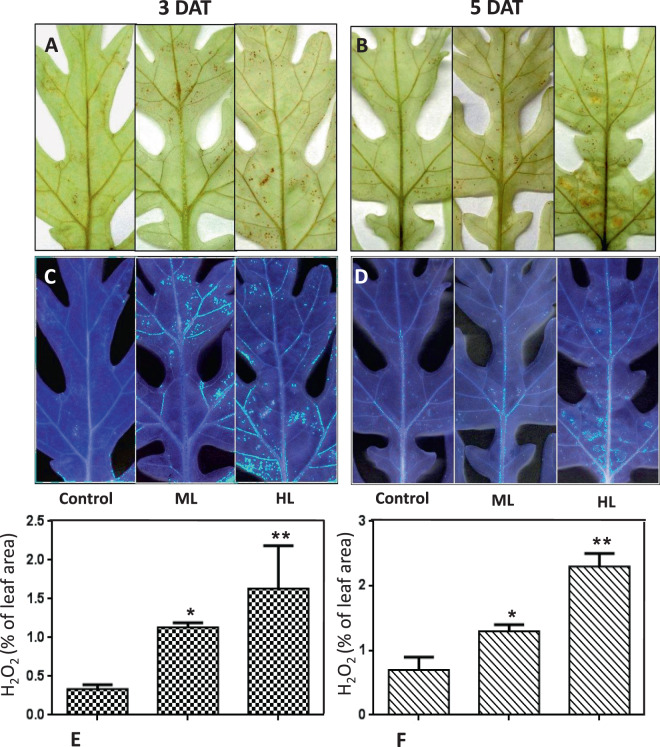
(**A**–**F**) *In situ* histochemical detection of hydrogen peroxide formed in *Parthenium hysterophorus* leaves under the effect of control (Control, 250 µmol photons m^−2^ s^−1^), moderate (500 µmol photons m^−2^ s^−1^) and high (1000 µmol photons m^−2^ s^−1^) light intensities at A. 3 DAT and B. 5 DAT. Quantification was performed for per cent H_2_O_2_ spots formed against total leaf area.

Accumulation of H_2_O_2_ was visually detected successfully in terms of H_2_O_2_-catalyzed polymerization of 3, 3-aminobenzidine (DAB) characterized by a brown precipitate (Fig. 2A,B). Accumulation of H_2_O_2_ was highest in the leaf after 5 DAT of HL followed by ML as compared to controls as well as 3 DAT of stressed plant.

A comparative analysis for estimation of amount of hydrogen peroxide generated under ML and HL was performed using image analyses software viz. ImageJ® (Fig. 2C,D). It was found that even in control plants there was generation of H_2_O_2_, 0.3% of total leaf area. Under ML it increased to 1.1% and was maximum under HL reaching 1.9% at 3 DAT. At 5 DAT, the amount of H_2_O_2_increased to 0.7%, 0.9% and 2.3% under control, ML and HL conditions, respectively (Fig. 2E,F). The increased leaf area of brown spots of H_2_O_2_ in ML and HL stress reflected direct connection with oxidative stress.

### More histochemical localization of superoxide (O2^−^) anions under HL stress

**Figure 3:**
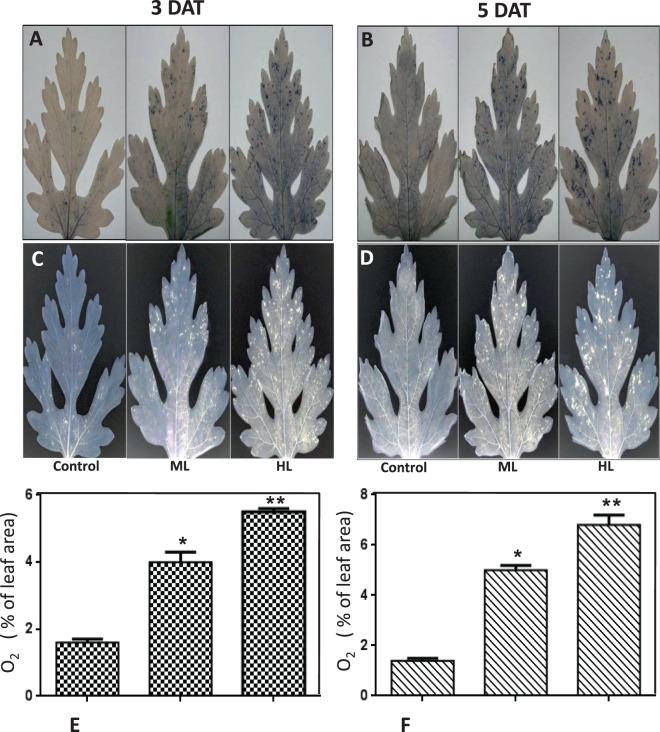
(**A–F**) *In situ* histochemical detection of superoxide radicals formed in *Parthenium hysterophorus* leaves under the effect of control (Control, 250 µmol photons m^−2^ s^−1^), moderate (500 µmol photons m^−2^ s^−1^) and high (1000 µmol photons m^−2^ s^−1^) light intensities at A. 3 DAT and B. 5 DAT. Quantification was performed for per cent superoxide radical (O_2_^−^) spots formed against total leaf area.

The main oxidant species viz. superoxide anion reduce nitroblue tetrazolium (NBT) to insoluble formazan. It was visualized as a dark-blue deposit. Several demarcated spots were observed in the leaf after 3 DAT of ML and HL stress. Spots increased in numbers and sizes after 5 DAT of light stress (Fig. 3A,B). Control plants at 3 DAT and 5 DAT also produced a few blue spots indicating generation of superoxide radicals at normal light conditions. Greater accumulation of superoxide was noted in HL conditions than in ML.

A comparative analysis of superoxide anions spot abundance under ML and HL was performed using image analyses software viz. ImageJ® (Fig. 3C,D). It was found that even in control plants there was generation of O_2_^−^ amounting to 1.4% of total leaf area under study. Under ML, it increased to 4.2% and was maximum under HL reaching 5.8% at 3 DAT. At 5 DAT, the amount of O_2_^−^ further changed to reach 0.9%, 4.9% and 8.3% under control, ML and HL conditions, respectively (Fig. 3E-F). Increased abundance of O_2_^−^ truly establishes the oxidative injuries at cellular locations in *P. hysterophorus*.

### Proline and total soluble sugar content

**Figure 4:**
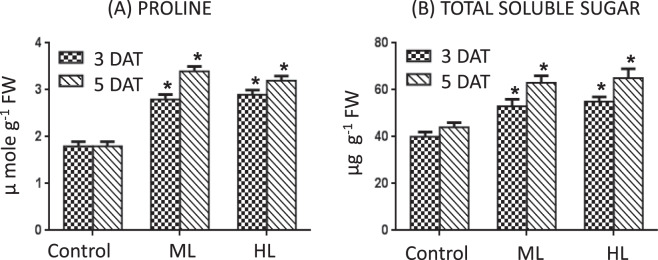
(**A,B**) Effect of moderate (500 µmol photons m^−2^ s^−1^) and high (1000 µmol photons m^−2^ s^−1^) light intensities on content of proline (**A**) and total soluble sugar (**B**) in leaf of *Parthenium hysterophorus* at 3 DAT and 5 DAT. Values are mean ± SE and n = 5.

Concentration of proline and total soluble sugar were found to be significantly higher in *P. hysterophorus* exposed to ML or HL, facilitating osmotic adjustment. Proline content did increase under both ML and HL stress. As compared to control plants, proline was 55% and 61% more at 3 DAT and 89% and 78% more at 5 DAT under ML and HL, respectively (Fig. 4A). Similarly, increase in total soluble sugar was also observed under both ML and HL stress. As compared to control plants, total soluble sugar accumulation was 33% and 38% higher at 3 DAT and 43% and 48% higher at 5 DAT under ML and HL, respectively (Fig. 4B).

### Total antioxidant capacity of leaf was higher under light stress

**Figure 5:**
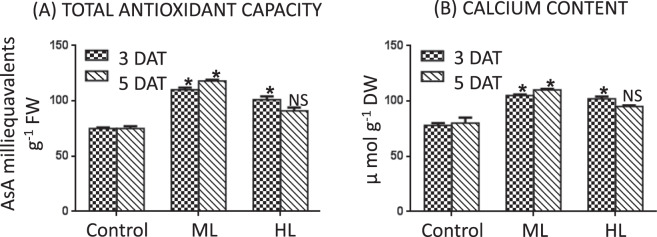
(**A,B**) Effect of moderate (500 µmol photons m^−2^ s^−1^) and high (1000 µmol photons m^−2^ s^−1^) light intensities on content of calcium (**A**) and total antioxidant capacity (**B**) in leaf of *Parthenium hysterophorus* at 3 DAT and 5 DAT. Values are mean ± SE and n = 5.

Total antioxidant capacity (TAC) of *P. hysterophorus* increased with increased duration of stress. However, *P. hysterophorus* exhibited maximum TAC in the ML treatment. As compared to control plants, TAC was 47% and 35% more at 3 DAT and 57% and 20% more at 5 DAT under ML and HL stress, respectively (Fig. 5A). Such finding indicates a substantial regulation of TAC as a defensive strategy of *P. hysterophorus* under ML and HL stress.

### Leaf calcium increased under light stress

An increase in content of calcium was observed under both ML and HL stress. As compared to control plants, calcium content was 28% and 25% higher at 3 DAT and 34% and 30% higher at 5 DAT under ML and HL stress, respectively (Fig. 5B). Due to its role in signal transduction, increase of calcium content might help *P. hysterophorus* in defense during ML and HL stress.

### Photosynthesis related parameters

#### Rate of photosynthesis (Pn)

**Figure 6:**
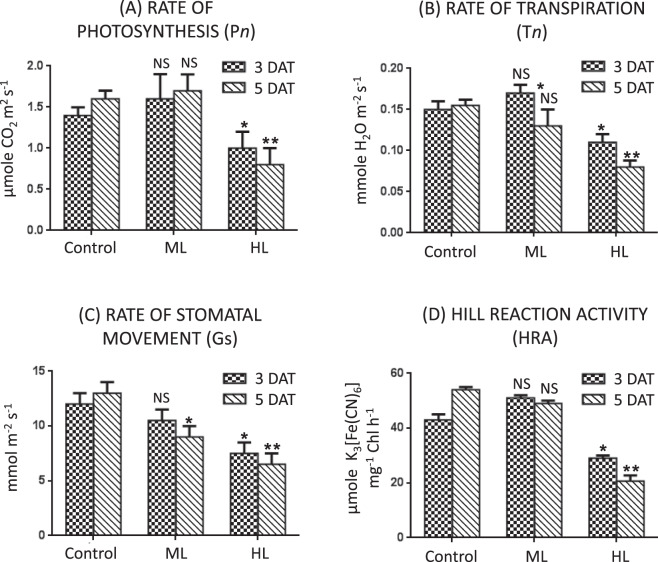
(**A–D**) Effect of moderate (500 µmol photons m^−2^ s^−1^) and high (1000 µmol photons m^−2^ s^−1^) light intensities on (**A**). rate of photosynthesis (P*n*)), (**B**) rate of transpiration (T*n*) and (**C**) rate of stomatal movement (Gs) in leaf of *Parthenium hysterophorus* at 3 DAT and 5 DAT. Values are mean ± SE and n = 5.

An increase in the rate of photosynthesis (P*n*) was observed under ML stress. However, this increase was notsignificant. Compared to the control plants, P*n* increased by 14% and 6% at 3 DAT and 5 DAT under ML stress, respectively. In contrast,a decrease was observed in HL stressed plants. When compared to control plants, P*n* decreased by 29% and 50% at 3 DAT and 5 DAT under HL stress (Fig. 6A). Results of this experiment suggested that the light responses in terms of photosynthesis were varied.

#### Light stress modulated rate of leaf transpiration (Tn), stomatal movement (Gs) and Hill reaction

Light stress affected the leaf transpiration (T*n*) values in *P. hysterophorus* plants due to light dependent opening and closing of stomata. As compared to control plants, T*n* values were increased by 13% and decreased by 19% at 3 DAT and at 5 DAT under ML stress, respectively. In HL stress, T*n* values were decreased by 26% and 50% when compared to controls at 3 DAT and 5 DAT, respectively (Fig. 6B).

A decrease in movement of stomata (G*s*) was observed under both ML and HL stress may be contributed by leakage of ions under stress conditions. As compared to control plants, G*s* decreased by 13% and 38% at 3 DAT and 30% and 50% at 5 DAT under ML and HL stress, respectively (Fig. 6C).

Activity of the Hill reaction was altered in both ML and HL stress conditions. Hill reaction activity was increased by 19% and decreased by 33% after exposure of ML and HL stress at 3 DAT, respectively. At 5 DAT of ML and HL stress, the activity of Hill reaction was reduced 9% and 61% while compared to controls, respectively (Fig. 6D). Results exhibit a link between Hill reaction and various light intensities.

#### Photosynthetic pigments affected by light stress

**Figure 7:**
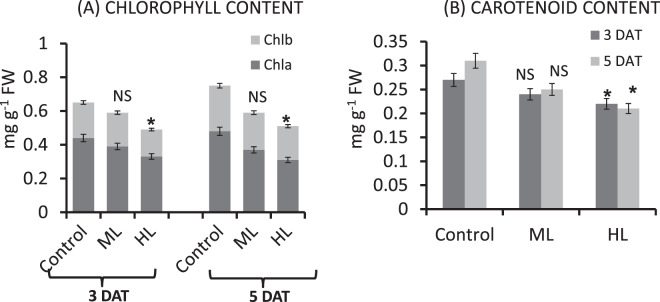
(**A,B**) Effect of moderate (500 µmol photons m^−2^ s^−1^) and high (1000 µmol photons m^−2^ s^−1^) light intensities on contents of Chl a, Chl b and total (a + b) chlorophyll and content of carotenoid in leaf of *Parthenium hysterophorus* at 3 DAT and 5 DAT. Values are mean ± SE and n = 5.

Both ML and HL stress led to a decline in the level of photosynthetic pigments including Chl a, Chl b and Chl total (a + b), and carotenoids exhibiting photosynthetic pigments sensitivity towards changes of light intensity. As compared to the control treatment, ML stress caused a decrease of 21%, 13% and 19% in Chl a, Chl b and total Chl, respectively at 3 DAT. At 5 DAT, the decrease was more severe reaching 27%, 22% and 25% in Chl a, Chl b and total Chl, respectively. In the HL treatment, decline in Chl a, Chl b and total Chl was 28%, 20% and 26%, respectively at 3 DAT. At 5 DAT, a further decline in the content of Chl a, Chl b and total Chl reached 40%, 33% and 38%, respectively versus the control (Fig. 7A).

As compared to control, a declinein the content of carotenoids14% at 3 DAT and 26% at 5 DAT occurred under ML stress. It declined 14% at 3 DAT and 32% at 5 DAT under HL stress (Fig. 7B).

#### Fresh weight and biomass also suffer decrease to light stress

**Figure 8:**
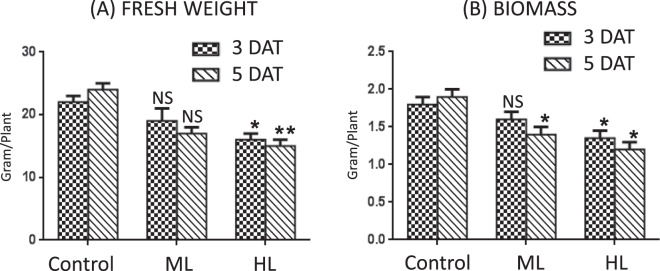
(**A,B**) Effect of moderate (500 µmol photons m^−2^ s^−1^) and high (1000 µmol photons m^−2^ s^−1^) light intensities on plant fresh weight (**A**) and dry weight (**B**) of *Parthenium hysterophorus* at 3 DAT and 5 DAT. Values are mean ± SE and n = 5.

Both ML and HL stress led to a decrease in plant fresh weight and dry weight (biomass) content. Fresh weight declined by 14% and 29% at 3 DAT and 5 DAT under ML stress, respectively. Under HL stress, reduction in fresh weight reached 27% and 42% at 3 DAT and 5 DAT, respectively (Fig. 8A). Dry weight declined by 11% and 26% at 3 DAT and 5 DAT under ML stress, respectively. Under HL stress reduction in dry weight reached 25% and 39% at 3 DAT and 5 DAT, respectively which showed reduced growth in HL stress (Fig. 8B).

#### *In-vitro* analyses of osmolytes accumulation on photon availability

In an *in vitro* experimental system, varied osmolyteconcentrations (0.1–1.0 M) were placed between an electronic light dependent resistor (LDR or photoresistor) and a light source (250 µmol photons m^−2^ s^−1^). Values of resistance were measured (208–225Ω). With increase in the osmolyte concentration, an increase in the resistance was noted (Table 1).

**Table 1:** Effect of increasing accumulation of osmolytes (Proline + Sucrose) on resistance (interference in photons transmission) measured using a light-dependent resistor (LDR).

S.N.	Osmolytes (M)		Resistance (Ω)
	Proline	Sucrose	
1.	0.0 (Water)	0.0 (Water)	208
2.	0.1	0.1	214
3.	0.2	0.2	218
4.	0.3	0.3	219
5.	0.5	0.5	223
6.	1.0	1.0	225

#### Alterations were induced in the chloroplast and mitochondrial ultra-structure under ML and HL stress

**Figure 9:**
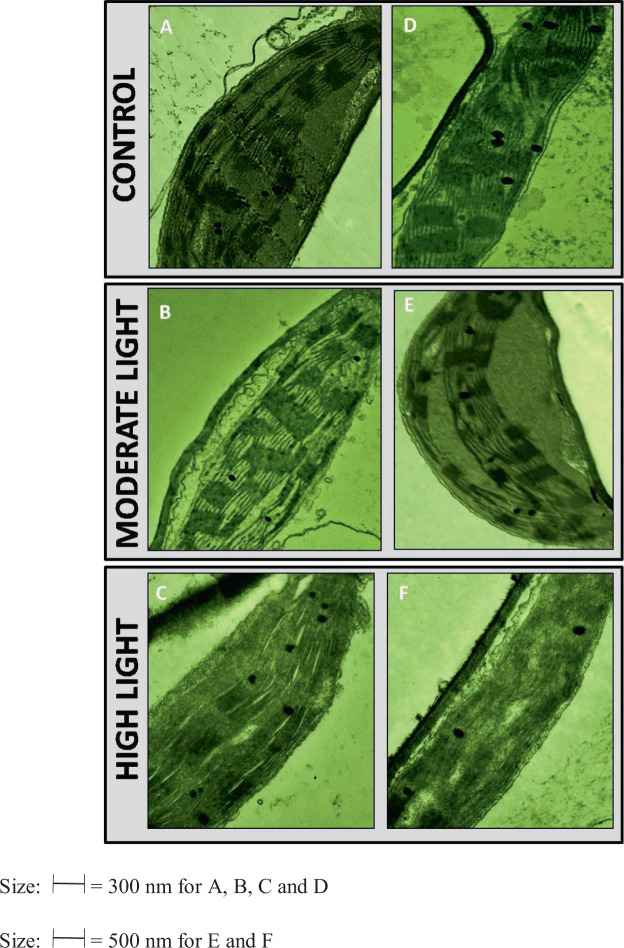
(**A**–**F**) Changes induced in ultrastructure of chloroplast by moderate (500 µmol photons m^−2^ s^−1^) and high (1000 µmol photons m^−2^ s^−1^) light intensities at 3 DAT and 5 DAT. A = Control (0 DAT), B = ML (3 DAT), C = HL (3 DAT), D = Control (0 DAT), E = ML (5 DAT), F = HL (5 DAT). 3 DAT and 5 DAT = 3 or 5 days after treatment.

The chloroplast of control plants showed regular elliptical shape with proper assemblies of frets, grana and envelope. In response to ML stress at 3 DAT and 5 DAT, little change was observed. Interestingly, as an early response (3 DAT) increase in density of grana was visible with the development of some desiccation-like symptoms. Abnormal swelling of chloroplast was noticed at 5 DAT. Plants were affected more under HL stress. At 3 DAT of HL stress grana had become flattened and diffuse forming a parallel network. Damage to the envelope was evident. Symptoms were severe at 5 DAT of HL. There was disintegration of the fret network exhibited spaces (Fig. 9A–F).

**Figure 10:**
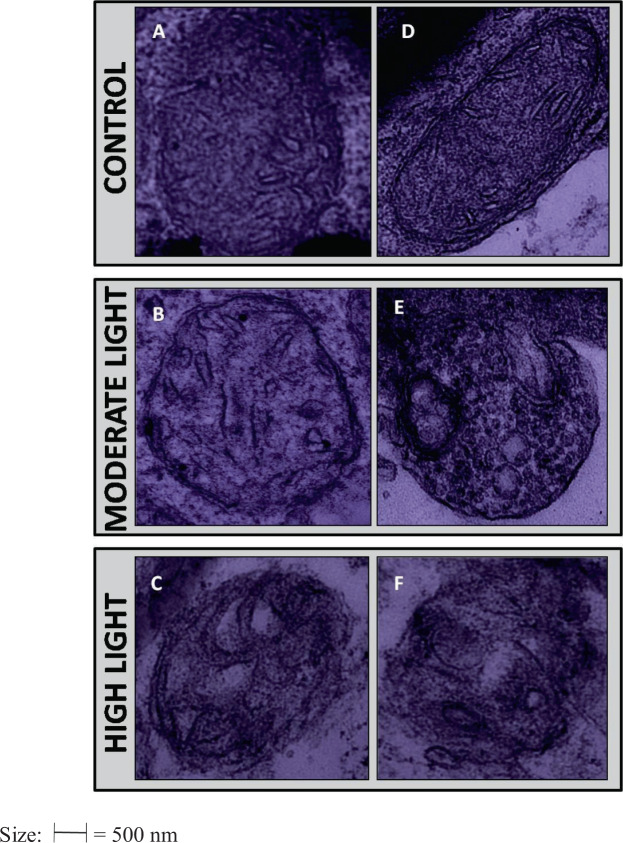
(**A**–**F**) Changes induced in ultrastructure of mitochondria by moderate (500 µmol photons m^−2^ s^−1^) and high (1000 µmol photons m^−2^ s^−1^) light intensities at 3 DAT and 5 DAT. A = Control (0 DAT), B = ML (3 DAT), C = HL (3 DAT), D = Control (0 DAT), E = ML (5 DAT), F = HL (5 DAT). 3 DAT and 5 DAT = 3 or 5 days after treatment.

The present studies also revealed changes in mitochondrial ultra-structure under ML and HL stress. Mitochondria of control plants exhibited oval or elongated shapes with a dense matrix and well-developed internal membranes. Under ML and HL stress, mitochondria showed with disorganization of matrix and internal membranes. Mitochondrial swelling and vacuolization were visible in both ML and HL stress conditions. It was prominent in the HL treatment at 3 and 5 DAT (Fig. 10A–F). Oxidative stress and generation of ROS were high in HL at 5 DAT, therefore, damage was more prominent in chloroplast and mitochondria under HL at 5 DAT than other treatments.

#### Changes in expression of proteins

**Figure 11:**
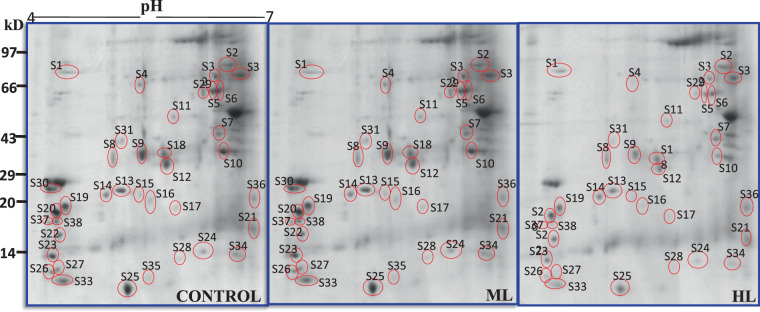
2-DE gel images representing proteins of interest selected for tryptic digestion based on differential expression in control versus moderate (500 µmol photons m^−2^ s^−1^) and high (1000 µmol photons m^−2^ s^−1^) light intensities exposed *Parthenium hysterophorus* at five days after treatments (5 DAT).

To further explore insights of physiological response of *P. hysterophorus* under ML and HL stress, leaf proteomes of *P. hysterophorus* were analyzed through two-dimensional electrophoresis (2-DE). In preliminary analyses maximum changes to the proteome were observed at 5 DAT. Therefore, proteomic characterization was made at 5 DAT. In this analysis, samples were in three replicates, and more than 120 protein spots were observed in every sample following staining. Of these, 38 protein spots showed increased (>1.2) or decreased expression (<0.67) in the treated samples (5 DAT) in relation to the control (0 DAT). In total, 38 differentially expressed proteins (Fig. 11) were identified successfully by MALDI-TOF-MS/MS analysis using the National Center for Biotechnology Information (NCBI) non redundant protein database (Table 2). The results depicted up-regulation than down-regulation of proteome in ML stress and vice-versa in HL stress. 21, 22 and 36 protein spots were up-regulated in both ML and HL stress treatments. The 3, 8, 15, 16, 28, 30, 32, 35, 37 and 38 protein spots were down regulated in both ML and HL stress treatments. These proteins represent candidate proteins which are associated with ML and HL stress responses in *P. hysterophorus*.

**Table 2:** List of modulated proteins identified in *P. hysterophorus* leaf. Spot IDs corresponds to the labeled 2D gels (Fig. 11).

Spot I.D.	Protein name	Plant species/Accession number	Exp.MW (kDa)/pI	Thr. MW (kDa)/pI	Gene name	Mascot score	Biological function
S1	Calcium dependent protein kinase 27	*Oryza sativa subsp. Japonica*/P53684	68/4.4	55.3/5.0	*CPK27*	78	Signal transduction
S2	Protein gamma response 1	*Arabidopsis thaliana/ Q9ZRT1*	73.01/6.7	67.7/6.9	*GR1 F4F15.230*	84	Response to DNA damage
S3	Probable LRR receptor-like serine/threonine-protein kinase	*Arabidopsis thaliana/* COLGH8	74/6.8	74.0/8.4	*F2K11.19*	73	Protein phosphorylation
S4	Proton pump-interactor 2	*Arabidopsis thaliana/* B3H4K7	63.5/5.3	67.56/6.4	*PPI2 K7L4.14*	59	May regulate plasma membrane ATPase activity
S5	Monodehydroascorbate reductase	*Arabidopsis thaliana/* P92947	55.3/6.4	53.5/8.1	*MDAR5 MDAR6*	77	Catalyzes the conversion of monodehydroascorbate to ascorbate
S6	Calcium-dependent protein kinase 6	*Arabidopsis thaliana/*Q38872	55.0/6.7	64.7/5.8	*CPK6*	82	Signal transduction
S7	Serine/threonine-protein phosphatase PP1 isozyme 3	*Arabidopsis thaliana/* P48483	34.4/6.6	36.87/5.6	*TOPP3 F22C12.20*	56	Protein dephospho-rylation
S8	Probable SAL3 phosphatase	*Arabidopsis thaliana/* Q8GY63	29.8/5.4	38.47/5.7	*SAL3 MBM17.9*	27	Signal transduction
S9	Glutathione S-transferase U4	*Arabidopsis thaliana/* Q9ZW27	31.9/5.2	25.93/5.4	*GSTU4 F16P2.16*	66	Detoxification role against ROS
S10	Protein brevis radix like −1	*Arabidopsis thaliana* gi/18407372	38.1/6.7	39.4/6.3	*BRXLI*	55	Regulator of cell proliferation
S11	Large subunit of ribulose-1,5-bisphosphate carboxylase/oxygenase	*Spinifex litoreus* gi/164565023	52.2/5.7	53.0/5.9	*rbcL*	93	Photosynthesis
S12	RNA pseudouridine synthase 1	*Arabidopsis thaliana/* Q7XA65	29.0/5.8	36.21/6.8	*F13N6.19 F14G9.4*	43	Response to hydrogen peroxide
S13	Calmodulin-like protein 1	*Oryza sativa/* Q8S1Y9	23.9/5.2	21.07/4.7	*CML1 Os01g0810300*	68	Calcium-binding protein that binds and activates CAMK1
S14	Glutathione S-transferase 1	*Triticum aestivum/* P30110	22.7/5.1	25.92/5.2	*GSTA1*	86	Detoxification of xenobiotics
S15	Auxin-responsive protein IAA7	*Oryza sativa/* Q6H543	22.0/5.4	32.41/6.3	*IAA7 LOC_Os02g13520*	54	Act as a repressors of early auxin response genes at less concentrations of auxin
S16	Basic leucine zipper 6	*Oryza sativa/* Q5JMK6	19.3/5.5	28.44/6.2	*BZIP06 LOC_Os01g55150*	28	Transcription regulation
S17	Probable WRKY transcription factor 74	*Arabidopsis thaliana/* Q93WU6	16.6/6.9	37.21/9.6	*WRKY74 F414.30*	56	Transcription factor
S18	Shikimate kinase 1	*Arabidopsis thaliana*/ Q9SJ05	31/5.7	34.18/7.6	*SK1 F7D8.26*	28	Phosphorylation of shikimic acid
S19	ATP-dependent Clp protease proteolytic subunit	*Chlorokybusatmophyticus/* Q19VC3	18.8/4.4	25.26/6.0	*CIpP*	32	Plays an important role in the degradation of misfolded proteins
S20	17.6 kDa class I heat shock protein	*Solanum peruvianum/* O82012	17.3/4.2	17.56/5.2	—	55	Stress response
S21	Photosystem I assembly protein ycf4	*Agrostis stolonifera/*AgstCp033	20.3/6.0	21.1/10	*ycf4*	46	Photosynthesis
S22	Probable calcium-binding protein CML15	*Oryza sativa/* Q6L4D4	16.4/4.4	21.32/5.2	*CML15 LOC_Os05g31620*	88	Potential calcium sensor
S23	Acyl carrier protein 2	*Arabidopsis thaliana/*O80800	15.0/4.1	14.21/4.8	*MTACP2 T8F5.6*	26	Carrier of the elongating fatty acid chain in fatty acid biosynthesis
S24	Desiccation-related protein	*Craterostigmaplantagineum/* P22241	15.1/6.1	16.3/5.9	—	62	Stress defense
S25	Calmodulin-like protein 7	*Arabidopsis thaliana/*Q9LNE7	13.1/5.1	17.05/4.3	*CML7 T21E18.4*	75	Potential calcium sensor
S26	Thioredoxin H4-1	*Oryza sativa/* Q9AS75	13.8/4.1	14.72/4.8	*Os01g0168200 LOC_Os01g07376*	84	Redox regulation of many cytosolic enzymes
S27	Calmodulin-1	*Arabidopsis thaliana/Q6LAE2*		5.8/4.2	—	93	Calcium ion binding
S28	Oxygen-evolving enhancer protein 2	*Arabidopsis thaliana* gi190358919	13.8/5.9	13.4/5.8	*PSBP1*	61	Photosynthesis Calcium ion binding
S29	Succinate-semialdehyde dehydrogenase	*Arabidopsis thaliana/*Q9SAK4	56.0/6.3	56.9/6.5	*ALDH5F1 SSADH1 T8K14.14*	23	Involved in plant response to environmental stress
S30	Peptide methionine sulfoxide reductase B4	*Arabidopsis thaliana/* Q9M0Z5	20.7/4.2	15.5/5.3	*MSRB4*	46	Plays a protective role against oxidative stress
S31	Polyamine oxidase 1	*Arabidopsis thaliana/*Q9FNA2	35.3/5.2	53.1/5.3	*PAO1, PAO MSH12.17*	27	Oxidation-reduction process, Polymine catabolic process
S32	Pentatricopeptide repeat-containing protein	*Arabidopsis thaliana/* gi15220337	66/6.4	91.1/8.1	*PCMP-H40*	31	mRNA modification
S33	Probable calcium-binding protein CML28	*Arabidopsis thaliana* /Q9SRP7	9.0/4.4	9.1/4.7	*CML28*	86	Probable calcium-binding protein
S34	Small subunit of ribulose-1,5-bisphosphate carboxylase/oxygenase	*Malus sp. (Crab apple)/*Q02980	14.3/6.8	20.5/9.0	*RBCS*	34	Photosynthesis
S35	Photosystem II protein J	*Dunaliellasalina* gi/246880733	4.3/5.8	4.32/8.3	*psbJ*	27	Photosynthesis
S36	Cyclic dof factor 4	*Arabidopsis thaliana/* O22967	19.1/6.9	19.31/9.3	*CDF4 DOF2.3*	25	Transcription factor
S37	Lactoylglutathione lyase	*Cicer arietinum gi*50214550	18.8/4.4	21.1/5.2		64	Catalyzes the conversion of hemimercaptal, formed from methylglyoxal and glutathione to Slactoylglutathione
S38	WPP domain containing protein 3	*Arabidopsis thaliana* gi/18421176	15.7/4.9	17.5/5.0	*WPP3*	48	Regulates mitosis

The 38 differentially expressed proteins were found to be related to a variety of physiological processes. Detail identified proteins including theoretical and experimental pI values, theoretical and experimental molecular weights, homologues protein/organism, encoding gene, cellular location and major functions have been provided in Table 2 and in supplementary Table S1. Gene Ontology (GO) terms showing enrichment have been represented in a pie chart (Figs. S1, S2 in supplementary material). These proteins were into GO categories: molecular function, biological process and cellular component.

**Figure 12:**
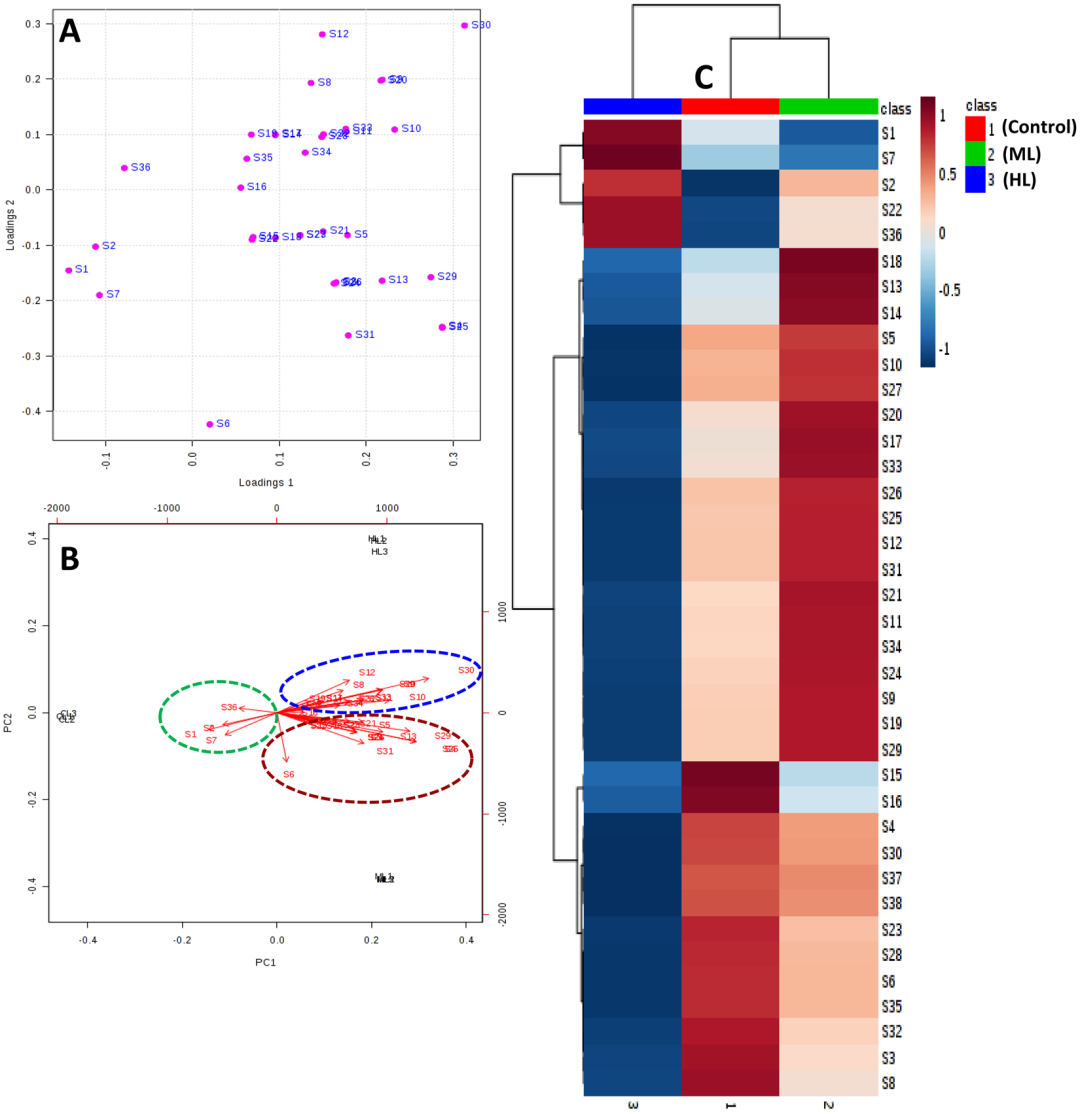
(**A**–**C**) Multivariate analysis of obtained data using MetaboAnalyst software. (**A**) Hierarchical clustering based on percentage volumes of the differentially modulated spots was performed. (**B**) Loadings plot displays a distinct partition of protein spots between control and stressed leaves. (**C**) PCA biplot shows the component scores and variable loadings obtained by PCA in two dimensions.

Hierarchical clustering was conducted to identify proteins with similar level of expression in the ML and HL stressed leaves (Fig. 12A). To normalize the scale of abundance, percentage volumes of every spot were subjected to log-transformation to base 2 before performing clustering using MetaboAnalyst software. Hierarchical clustering distinguished the abundance patterns of the differentially modulated abundance in protein spots (Fig. 12A). Unsupervised PCA loadings were used to visualize the data and compare the differentially modulated proteins obtained from the two-dimensional gels of ML and HL stressed leaves (Fig. 12B,C).

The first principal component axis (PCA1) explained 93.5% of the variation for the total values of proteome change under light stress in leaf samples of *P. hysterophorus*. The second PC axis (PCA2) had lower significance (6.4% of variation). Calcium dependent protein kinase 27 (S1), protein gamma response 1 (S2), serine/threonine-protein phosphatase PP1 isozyme 3 (S7) and cyclic dof factor 4 (S36) had the maximum contribution to the second axis. The length, direction and angle between the vectors indicate the strength of the vector effect and correlation between vectors. Long vectors for all proteins show that the vector significantly influenced the results of the analysis. It was clear that the putative SAL3 phosphatase (S8), putative mannitol dehydrogenase (S10), RNA pseudouridine synthase (S12), basic leucine zipper 6 (S16), putative WRKY transcription factor 74 (S17), oxygen-evolving enhancer protein 2 (S28), peptide methionine sulfoxide reductase B4 (S30), probable calcium-binding protein CML28 (S33) and small subunit of ribulose-1,5-bisphosphate carboxylase/oxygenase (S34) grouped together with positive loading on the right upper side of the biplot. This suggested that these proteins were positively correlated with each other. Proton pump-interactor 2 (S4), monodehydroascorbate reductase (S5), calcium-dependent protein kinase 6 (S6), calmodulin-like protein 1 (S13), photosystem I assembly protein ycf4 (S21), putative calcium-binding protein CML15 (S22), calmodulin-like protein 7 (S25) and polyamine oxidase 1 (S31) were observed on the right lower side of the biplot indicating an association to each other. On the biplot, calcium dependent protein kinase 27 (S1), protein gamma response 1 (S2), serine/threonine-protein phosphatase PP1 isozyme 3 (S7), cyclic dof factor 4 (S36) segregated in opposite orders indicating that the expression level of these proteins are negatively correlated. The angles between different antioxidants were small, indicating that these were positively correlated. Proteins present in blue and brown circles had small angles, indicating that these proteins were positively correlated in their respective groups.

#### Interaction Network Analysis

**Figure 13:**
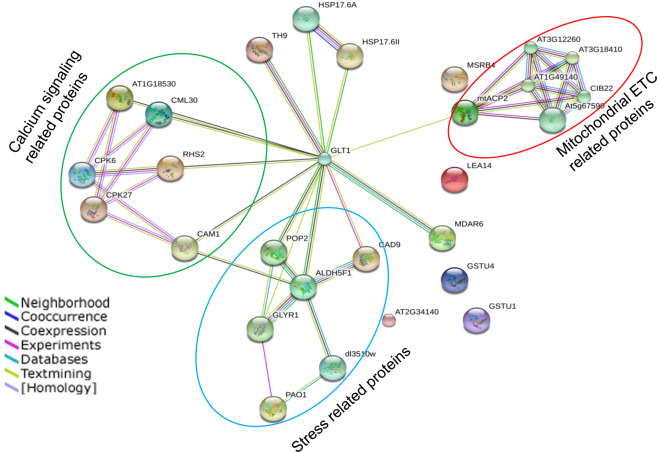
Communication network analysis of the identified proteins using STRING software. Different colored lines depict different types of evidences for the association of the proteins. The circled nodes are the important networks under ML and HL stress.

Proteins interact together within cellular networks, rather than working in an isolated way^19^. The STRING database describes potential of protein–protein interactions, involving indirect (functional) as well as direct (physical) associations. A network of protein interactions for differentially expressed proteins is shown in Fig. 13. Off the many interacting proteins, the calcium signaling related proteins and proteins associated with mitochondria (electron transport chain, ETC) both represented the highest proportion (21.4% of each categories). More importantly, acyl carrier protein 2 (spot 23) and calmodulin-like protein 7 (spot 25) were interacting proteins within the network, indicating that calcium signaling and mitochondrial ETC were of the utmost importance for the response to ML and HL treatment in *P. hysterophorus*.

## Discussion

*P. hysterophorus*is a weed plant that can easily adjust and grow successfully under adverse environmental conditions^17,20^. In this study, exposure of *P. hysterophorus* to moderate light (ML, 500 µmol photons m^−2^ s^−1^) and high light (HL, 1000 µmol photons m^−2^ s^−1^) stress induced notable changes to physiological, photosynthetic parameters, proteomic profiles and growth in relation to the plants grown under normal light (Control, 250 µmol photons m^−2^ s^−1^). The results provided evidence of *P. hysterophorus* achieving tolerance by common and contrasting responses to ML and HL treatments. These responses provide insight into the tolerance mechanisms against high light intensities operating in *P. hysterophorus*.

The degree of ML and HL used in the present study has previously proved to be highly deleterious to other plants used as models in earlier studies^21,22^. In this study, to our surprise, the magnitude of oxidative stress was low in *P. hysterophorus*. However, HL induced higher level of oxidative stress as compared to ML. The reason for higher oxidative stress might be more photooxidative damage to biomolecules might be caused by increased generation of reactive oxygen species (ROS). Both ML and HL could induce formation of more hydrogen peroxide and superoxide radicals which was evident by histochemical localization. HL induced high levels of ROS compared to ML indicating less-sensitivity of *P. hysterophorus* to ML. It can be concluded that this plant is better equipped to limit the generation of ROS and this helps in explaining of tolerance to ML.

Photosynthetic pigments are necessary for light harvesting and in production of reducing powers for running Calvin cycle and performing other metabolic processes. Both chlorophyll a as well as chlorophyll b are vulnerable to drought and HL^23^. A decrease in chlorophyll a and chlorophyll b has been observed in some studies published earlier using various plants^11,24,25^. The ML and HL-induced changes in the leaf chlorophyll content might be attributed to disrupted biosynthesis or enhanced degradation of pigments. Time-dependent responses of photosynthetic pigment to ML and HL stress were observed in the present study. The reduction was more pronounced with increased HL duration. The lower Chl (a + b) at a high stress level also indicate stress damage to the photosynthetic apparatus showed in present study (Fig. 9A–F). Carotenoids as photosynthetic pigments have further roles and these can help plants combat ML and HL stress. They are needed for the photo-protection of photosynthesis and have been found to play a major role as a precursor in the synthesis of signaling molecules. Our results of carotenoid contents in ML stress are consistent with those of Hazrati *et al*. (2016) who showed an increase in carotenoid level in *Aleo vera* under light stress.

Plant gas exchange is very important for biomass production. In the present study, photosynthetic rate (P*n*), stomatal conductance (G*s*) and transpiration rate (T*n*), were found to decrease due to stress in all treatments and most significantly at 5 DAT. Stomatal closure known to occur due to higher levels of ABA produced by plants under light stress^26^. This decrease in stomatal conductance leads to a marked reduction in other gas exchange parameters such as transpiration rate and photosynthetic rate. Stomata modulate the CO_2_ entry for the photosynthesis and the exit of the water vapor which is well documented.

Physiological interruptions in plants induced by light stress are also marked by changes in the ultrastructure of cell organelles. These impacts are reflected in the stability of grana and envelope^27^. In the present study, both ML and HL modified the structure of chloroplasts. Under ML stress, grana were more compact indicating the adaptive feature of chloroplast for this plant. However, under ML stress the overall structure was little altered perhaps because this plant has already curtailed stress effects by accumulation of compatible solute proline and total soluble sugar. Flattening of lamellae damage to envelope under HL stress might be explained by a greater degree of plasmolysis. However, this modulation the chloroplast structure might be due to changes in the fatty acid profile of thylakoid membranes as observed under salinity stress^69^. On the other hand, elongated mitochondria were observed under control conditions that might be undergoing division to maintain continuous ATP production. Spherical and rounded mitochondria were seen under ML and HL stress. The mitochondrial shape influences their movement ability inside the cell. Elongated mitochondria are found to be more movable in nature. Energy status of cell is closely related to the mitochondrial shape and their mobility^28^. Previous reports have suggested that the dynamism of mitochondria shape is related to calcium signaling^29,30^ which is consistent with the proteome findings in the present study.

The Hill reaction, and associated PSII responses, are more stress-sensitive than PSI^31^. The site of the Hill reaction sensitive to light is the oxygen-evolving enhancer protein (OEE, S28) complex system^32^. Hill reaction activity was changed non-significantly in the ML treatment and decreased significantly in response to HL stress suggesting that *P. hysterophorus* possibly maintained better PSII function in ML than under HL stress, a conclusion supported by our proteomic study (S28).

A further adaptive feature in response to ML and HL stress was the elevated accumulation of proline under ML and HL stress. Proline is believed to act as a ROS scavenger, an osmolyte and a molecular chaperone hence leading to stabilization of the protein structure, thus keeping the cells safe from damage occurring through stress^33^. The treatment of *Parthenium* plants with ML and HL stress increased proline content both at 3 DAT and 5 DAT. The maximum proline accumulation was recorded in ML stressed *P. hysterophorus* at 5 DAT. Higher proline levels suggest that compatible solutes contribute to the cellular osmoregulation and detoxification of reactive oxygen species^34^. There was also an increase in total soluble sugars under ML and HL stress at 3 DAT (Fig. 4B). The high level of sugars (osmo-regulators)^35^ and the higher content of proline in HL and ML stress conditions may might play an important protective role to savor *P. hysterophorus* against high light intensities.

Present study hypothesized a possible condition that might occur in the surrounding of chloroplast with respect to different osmolytes concentrations. Would such changes in solute concentration anyhow influence the availability of photons at the photosystems? The answer came from an experiment where a such a ‘probable *in vivo* condition’ was established *in vitro*. It was seen that photon availability/activity supported the hypothetically set notion (Fig. 1). The results indicate that under stress conditions accumulation of osmolytes might result in lesser availability of photons at the chloroplast. This, in turn, might help in lesser accumulation of energy inside chloroplast or helping in the energy dissipation. It is speculated that higher accumulation of osmolytes in *P. hysterophorus* leaves under ML or HL stress could result as a barrier between light photons and chloroplast/thylakoids. Such a possibility might occur in green cells of plants which may lead to inhibition of photolysis of water, evolution of electrons and synthesis of reducing power and ultimately causing a reduction too in photosynthesis of plants.

The understanding of role of Ca^2+^ as a player at the global level in the plant under light stress is not clear. As a secondary cellular messenger, Ca^2+^ do serves as a direct indicator of cellular changes under extracellular stimulus in view of the changes seen in its concentration^36^; but how about affected overall metabolic and proteomic scenario? A number of clues were obtained through present study. In this study, progressive increase in calcium content was noted at 3 and 5 DAT as compared to the control. However, rise of calcium at 3 DAT was much higher. This observation suggests that the plant initially requires high concentration of Ca^2+^ for the induction of other physiological pathways which are involved in stress tolerance. Proteins related to Ca^2+^ including CaM/CML/CPKs have been widely investigated and are clearly involved in stress signaling of plants^37^. Our results showed that the modulation in the abundance of Ca-associated proteins near could be a part of stress tolerance in *P. hysterophorus*.

*P. hysterophorus* produced more antioxidants in both stress conditions indicated by enhanced total antioxidant capacity (TAC) to control and stress-induced oxidative damage. The antioxidant activity is derived from endogenous bioactive compounds. *P. hysterophorus* belongs to weed plant which is rich of endogenous bioactive compounds^38^. The occurrence of diverse nature of such molecules in this plant might execute total antioxidant capacity^18^. In total, it is very evident that light-stressed *P. hysterophorus* had a metabolome that could counter higher level of oxygen species as it does under other stress^70^.

The leaf proteomic study identified 38 protein spots; showing significantly higher differential expression in HL and ML-stressed *P. hysterophorus* (Table 2), which might specifically play important role(s) against HL and ML stress and help in raising tolerance in *P. hysterophorus*.

We observed a large proportion of the proteins whose abundance changed significantly under stress were associated with calcium signaling. This was an important clue towards the role of calcium and calcium-related proteins against the high light stress. In the quantitative proteomic analyses, 7 of the identified proteins were related to calcium signaling. It is very well-known fact that intracellular changes in calcium ions (Ca^2+^) related to stress are detected by sensor proteins calmodulin (CaM) and calmodulin-like protein (CML) in the plant cell^39^. Calmodulin is involved in mediating the control of several ion channels, enzymes, generation of reactive oxygen species, metal ions uptake, and modulation of transcription factors^40^. In our study, three proteins calmodulin-like protein 1 (S13), calmodulin-like protein 7 (S25) and calmodulin-1 (S27) were up-regulated in ML stressed plants as important calcium sensors. Similarly, activities of CaM-binding proteins have also been associated with stress responses in this study. Such CaM-binding proteins like probable calcium-binding protein CML15 (S22) was over-expressed in ML and HL stress while probable calcium-binding protein CML28 (S33) was up-regulated in ML and down-regulated in HL stress. Calcium-dependent protein kinases (CPKs) are also significant in stress responses which convert calcium signals into phosphorylation events. The calcium-dependent protein kinase 27 (S1) and calcium-dependent protein kinase 6 (S6) were differentially expressed in both stresses. These changes in kinase suggest coordinated contribution to phosphorylation signaling in response to ML and HL stress. Activities of proteins related to calcium are modulated in such a manner that it could provide versatility to the stress-associated signal transduction pathways, which allows tolerance in *P. hysterophorus* by maintaining homeostasis between calcium and various cell related processes.

In the ML and HL treated *P. hysterophorus* leaves, several proteins which are directly associated with stress response were identified. Abundance of 17.6 kDa class I heat shock protein (S20) increased under ML and decreased in HL stress. Small heat shock proteins have been found to play a big role in the tolerance mechanism to stress through preventing the aggregation of denatured proteins and protecting them^41^. Earlier reports conducted on Arabidopsis and Peanut showed that sHSPs do increase in HL and heat stress^42,43^. Additionally, desiccation-related proteins (S24) play a major role in conferring stress tolerance^44^. Our results demonstrate enhanced expression of this protein which might be linked to minimizing the oxidative stress generated due to ML. However, it was decreased in HL stress.

Antioxidant enzymes regulate the cellular level of reactive oxidative species (ROS) generated during the essential metabolic processes or through oxidative stress metabolism^15^. Thus, antioxidant capacity significantly affects the degree of plant tolerance against the stress. Glutathione S-transferases (GST) (S9), (S14) were more abundant in ML and less abundant in HL-stressed *P. hysterophorus*. On the other hand, monodehydroascorbate reductase (S5), an essential component of glutathione-ascorbate cycle and thioredoxin (S26) that reduces disulphide bonds and donate electrons to various antioxidant enzymes were up-regulated in ML stress^45^. These findings indicated not only the universal functions of such proteins in *P. hysterophorus* but also the vital roles of some proteins with higher fold changes in the improved ML tolerance in *P. hysterophorus*. Though, abundance of these proteins was slightly decreased or just maintained, might be helping in combating HL stress.

Transcriptional-regulating genes are known to play a major role in generating plant response under stressed conditions^46,47^. Expression modulation of such genes in response to ML and HL could be detrimental to plant salvage against stress. Previous studies have shown that WRKY transcription factor mediate the regulation of gene expression, which plays a crucial role in response to abiotic stress^48^. In our data, the abundance of WRKY 74 (S17) in ML and HL treated samples was differential hence demonstrating the role of WRKY 74 in the ML and HL stress responses. Many physiological processes of plants regulated by DNA-binding one zinc finger (Dof) (S36) protein, which was over-expressed in ML and HL stress, suggesting that plant maintains physiological events under stress. Moreover, auxin responsive protein IAA 7 (S15) showed less abundance in ML and HL stress indicating that *P. hysterophorus* makes efforts to resume cell division and proliferation during ML and HL stress.

Light intensity is an important factor of photosynthesis that directly and indirectly affects this primary process of plant. RuBisCO is a key enzyme of photosynthesis which catalyzes the conversion of inorganic carbon to the organic carbon^49^. In the present study, RuBisCO (S11, S34) has higher abundance under ML and lower under HL stress. PSI and PSII also participate significantly in photosynthetic adjustment to the environmental stresses by regulating electron flow^50^. Furthermore, photosystem I assembly protein ycf4 (S21) is concerned associated the assembly of the photosystem I complex, which is the part of an energy-harvesting process and found more abundant in ML and less abundant in HL intensities. Similarly, photosystem II protein J (S35) is a component of PSII core complex showed same expression pattern under ML and HL stress. The OEE1, OEE2, and OEE3 are the subunits of oxygen-evolving enhancer protein (OEE) which are involved in photolysis of water mediated by PSII. The OEE2 protein was easily removed from the PSII under stress conditions^15^. Under-expression of OEE2 (S28) in ML and HL stress indicates, PSII is a more sensitive target of stress. Apart from this, proteins related to calcium signaling and those involved in photosynthesis were also more active part of proteome as compared to other group of proteins suggesting to play important role in *P. hysterophorus* vitality under light stress conditions.

Under changing low light conditions photosynthetic

In our study, we identified a proton pump interactor 2 (S4) that controls the proton pump mediated activity of the cell membrane ATPase^51^, which was found to be less abundant in HL and ML-induced stress. The formation of succinate from succinate semialdehyde, catalyzed by succinate semialdehyde dehydrogenase increases the accumulation of succinate and GABA content^52^. Higher abundance of succinate semialdehyde dehydrogenase (S29) as a response to ML stress and decreased in HL-induced stress was noted. This could be an adaptation to keep synthesizing NADH amid stressful conditions for sustenance of plant. Also, polyamine oxidase 1 (S31) was found to be more abundant in ML and less abundant in HL stressed *P. hysterophorus*. Such differential expression of polyamine oxidase 1 might affect the metabolism of polyamines and hydrogen peroxide. Thus, to challenge ML and HL-induced stress in leaf tissues, changes in profile of proteome is necessary either through increased abundance and by lowering the abundance of many other proteins. This modulation of proteome profile could be associated with maintenance of growth and development and at the same time tackling the high light stress. These proteomic players are probably responsible for making *P. hysterophorus* a global weed.

**Figure 14:**
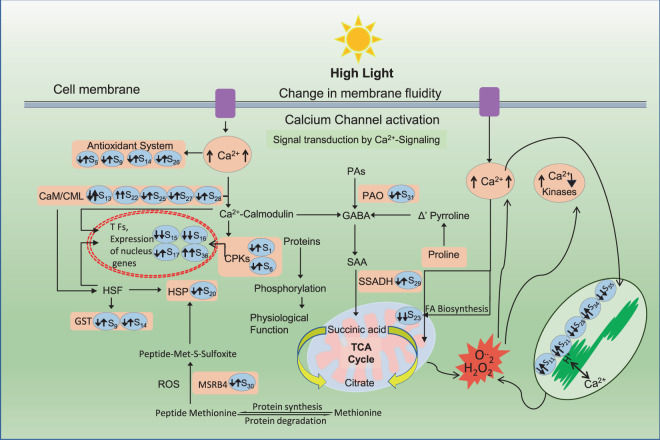
An outline of metabolic pathways of *P. hysterophorus* modulated under ML and HL stress. Up-ward arrow indicates over-expressed proteins and downward arrow indicates under-expressed proteins in ML and HL stress. S denotes protein spot on 2D gels.

On the whole, this study put together chloroplast and mitochondria ultrastructural, biochemical, and proteomic response of *P. hysterophorus* to moderate (ML) and high (HL) light intensities. Our data provide important information on key proteins and metabolic pathways involved in the response of *P. hysterophorus* to light stress (Fig. 14).

ML, increased level of calcium ions as indicated up-regulation of calmodulin-like protein 1, 7 /CML 28, 15 / calcium dependent protein kinase 6. These proteins might assist up-regulation of glutathione S-transferase, WRKY and heat shock protein. In contrast, proteins related to calcium signaling were low in abundance under HL stress. *P. hysterophorus* upholds the photosynthesis during ML stress through over-expression of rubisco and photosystem I assembly protein ycf4. Photosynthesis was repressed in HL stress might be due to inhibition of oxygen-evolving enhancer protein 2 and rubisco. However, cyclic dof factor 4 and protein gamma response were increased in both ML and HL stress. They probably help to sustain physiological process in *P. hysterophorus* under light stress. A comparison of biochemical, physiological and photosynthesis related parameters after three and five days of light stress treatment suggested that *P. hysterophorus* is more tolerant to ML than HL stress. *P. hysterophorus* accumulates soluble sugar and proline to maintain physiological balance; however, such accumulation could be a barrier between light photon and thylakoids as reflected by decreased photosynthesis in both ML and HL stresses. Acyl carrier protein 2 (ETC) and calmodulin-like protein 7 played a central role in communication with other proteins induced by light stress described by our PPI network analysis. Thus, our study elucidates about major strategies of *P. hysterophorus* involved in the adaptation toward light stress.

## Methods

### Plant material and growth conditions

Seeds of *Parthenium hysterophorus*, collected from the plants cultivated in culture room at Department of Biotechnology of Jamia Millia Islamia (A Central University), New Delhi. Plants of *Parthenium hysterophorus* were validated by Botanical survey of India Dehradun, India and cultured under control condition in a plant growth chamber as mentioned in Ahmad *et al*.^15^. The seeds were harvested at the maturation and used in the present study. Around 100 seeds were surface-sterilized using 0.3% KMnO_4_ for a period of 10 mins, followed a thorough rinsing by distilled water for ten times. One seed per pot (6” × 6”, filled with 300 g Soilrite®) was germinated and grown for 30 days. Plants were grown under controlled conditions (65% relative humidity, 10 h photoperiod, and 35 °C light/20 °C dark). Fully expanded mature leaves were harvested at 3 and 5 days after treatments (DAT). The plants were supplied with half-strength Hoagland nutrient medium^53^ on alternate days as per water holding capacity (WHC) of the soil.

### Treatments

Thirty-days-old plants were split into three sets viz. control (250 µmol photons m^−2^ s^−1^), moderate light (ML, 500 µmol photons m^−2^ s^−1^) and high light (HL, 1000 µmol photons m^−2^ s^−1^) treatments. The HL and ML treatments were given for 3 h (12 am to 3 pm) for 3 d and 5 d.

### Thiobarbituric acid reactive substances (TBARS) estimation

The magnitude of oxidative stress was estimated in terms of thiobarbituric acid (TBA) reactive substances (TBARS) by the method of Heath and Packer^54^. Fresh leaf tissue was homogenized in 1% (w/v) TCA, vortex-mixed and centrifuged at 6,708 × g for 10 min. One mL aliquot was mixed with 4 mL of 0.5% (w/v) of TBA and heated at 99 °C for 30 min. Mixture was immediately cooled and centrifuged at 2,795 × g for 5 min. Absorbance of supernatant was read at 532 nm and 600 nm. Unit of TBARS was expressed in nmoles g^−1^ FW.

### Histochemical detection of hydrogen peroxide and superoxide radicals

The accumulation of hydrogen peroxide (H_2_O_2_) and superoxide (O_2_^−^) anion in leaves of *P. hysterophorus*was histo-chemically visualized using nitroblue tetrazolium (NBT) and 3,3′-diaminobenzidine (DAB) staining, respectively, by the method as mentioned in Scarpeci *et al*.^55^.

### Visualization of hydrogen peroxide

Leaves were placed for overnight in DAB solution (1 mg mL^−1^, pH 3.8). Leaves were then boiled in ethanol for 10 min to remove chlorophylls. Numerous dots of reddish-brown color were visible on the leaves, depicting presence of hydrogen peroxides. Leaves with visualized hydrogen peroxide spots were digitized.

### Visualization of superoxides

Leaves were float on a solution [50 mM sodium phosphate (pH 7.5) and 0.2% (w/v) NBT] for 2 hours. Number of dark-blue spots was observed in the leaves depicting the formation of the insoluble formazan compound obtained by the reaction of NBT with superoxides.

### Proline content

Method of Bates *et al*.^56^ was used for estimation of leaf proline content. Half gram of fresh tissue was homogenized in 10 mL of 3% (w/v) sulphosalicylic acid. Mixture was spun at 5590 × g for 10 min. In 2 mL of aliquot, equal amount of acid ninhydrin and glacial acetic acid were added. Mixture was boiled in a water bath at 100 °C for 30 min. Reaction was stopped in an ice bath followed by addition of 4 mL of toluene. An intense vortex-mixing was performed and after settlement upper pinkish-red layer (toluene) was read at 520 nm. A standard curve of L-proline was used as standard. Proline concentration was expressed as μmol proline g^−1^ FW.

### Estimation of calcium content

Calcium content in the leaves was estimated as mentioned in Masson *et al*.^57^ using inductively coupled plasma mass spectrometry (ICP-MS) (Agilent, USA). Leaf samples were dried in a hot-air oven at 70 °C for 48 h. 100 mg dried leaf sample was ground and digested in acid mixture of HNO_3_ and HClO_4_ in ratio of 5:1 (v/v). Samples were analyzed and estimated against the standard curve of calcium.

### Total soluble sugar content

Total soluble sugar content was estimated by the method of Irigoyen *et al*.^58^. Half gram fresh leaf was homogenized with 80% (v/v) ethanol. Homogenate was centrifuged at 2415 × g for 15 min. Supernatant (0.1 mL) was added with 12.5 mL of 80% (v/v) ethanol and 1 mL of 0.2% (w/v) anthrone. Mixture was incubated on ice for 5 min and absorbance was read at 620 nm. Total soluble sugar content was calculated against standard curve of glucose and units were expressed as µg g^−1^ fresh FW.

### Total antioxidant capacity (TAC) estimation

One gram of leaf sample was homogenized using 80% (v/v) ethanol under cold condition. Mixture was centrifuged at 13,148 × g at 4 °C for 10 min. Antioxidant capacity of ethanolic extracts of *P. hysterophorus* was measured with phosphomolybdenum using of ascorbic acid as the standard in 1 mL of TAC reagent (3.3 ml H_2_SO_4_, 78.416 mg ammonium molybdate and 335 mg sodium phosphate in 100 ml final volume made by distilled water), as per the method of Prieto *et al*.^59^. The mixture was incubated within a water bath set at 95 °C for a span of 90 min. The absorbance was measured at 695 nm. The values were expressed as milli equivalents (mEq) of ascorbic acid per litre (AsA/L).

### Chlorophyll content

Estimation of the chlorophyll content (chla + chl b) was done by Hiscox and Israelstam^60^ method and calculated using standard formulae^61^. Extraction of fresh leaves (0.1 g) was done in 8 mL of dimethyl sulfoxide (DMSO) at 65 °C for 1 h. Final volume was made to 10 mL and the absorbance read at 480 nm, 645 nm, 520 nm and 663 nm. Subsequently, contents ofchl a, chl b, chl total (a + b) and carotenoids were estimated to expressed as mg g^−1^ FW.

### Ultrastructure of chloroplast and mitochondria

Ultrastructures of chloroplast and mitochondrial were studied as per the method mentioned in Qureshi *et al*.^27^. Fresh leaf was taken and cut to 1–3 mm^2^ pieces followed by its storage in fixing solution consisting of 2% paraformaldehyde, 1% formaldehyde and 2.5% glutaryldehyde. Samples were subjected to vacuum infiltration for 10 mins and placed at 4 °C overnight. Using 0.1 M phosphate buffer (pH 7.4), the samples after washing were kept in osmium tetraoxide for 2 h at 4 °C and once again washed using 0.1 M phosphate buffer (pH 7.4). Dehydration of samples was done using 30–90% acetone and then in dry acetone (saturated with copper sulphate) for 1 h at 4 °C. Toluene treatment was given to the samples for 1 h twice and then kept in toluene and resin (3:1) mixture for 1 h in vacuum, following which impregnation was done in resin and toluene (2:2 and then 3:1) mixture. Finally, samples were subjected to impregnation in pure resin at room temperature for 6 h. 500 nm of sections were cut and staining was done for 20–40 seconds using 1% methylene blue. Grids of 60–90 nm in size were made and staining was done using heavy metal solution, lead citrate and uranyle acetate. Then the sections were visualized under transmission electron microscopy (TEM, Model H-800, Hitachi, Japan) for studying chloroplast and mitochondria ultrastructure.

### Photosynthesis related parameters

With the use of GFS-3000 portable photosynthetic system (Walz, Germany), the *in vivo* net photosynthetic rate (P_n_) was determined on a fully expanded 3^rd^ leaf from the top, with light saturating intensity as mentioned by Bashir *et al*.^62^. Determination of photosynthetic rate (P_n_), rate of transpiration (T_n_) and stomatal conductance (G_s_) was done using saturated pulses (600 ms) of white light (4500 µE m^−2^ s^−1^). All the measuring was conducted in between 10:00 and 11:00 AM, with about 80% of relative humidity, 22–24 °C of leaf temperature and 355 mmol mol^−1^ of ambient carbon dioxide concentration.

### Hill Reaction activity

Chloroplast isolation and determination of Hill reaction activity: The leaves of *P. hysterophorus*were processed to isolate chloroplastsin 0.05 M Tris-HCl buffer, with pH7.6, consisting of 0.4 M sucrose along with 0.01 M NaCl as described by Yang *et al*.^63^ with some slight modification. Extraction buffer was used twice to wash the chloroplasts and eventuallythe chloroplasts were suspended in the extraction buffer. The Hill reaction activity of thechloroplasts which were isolated was measured by the rate of photoreduction of K_3_[Fe(SCN)_6_] as described by Yang *et al*.^63^. The reaction mixture (final volume 3 ml) consisted of 50 mM Tris-HCl buffer (pH 7.6), 5 mM MgCl_2_, 2 mM K_3_[Fe(SCN)_6_] nd chloroplast preparation having approximately 20 μg of chlorophyll. The tubes of the reaction mixture were subjected to illumination at 300 μmol m^−2^ s^−1^ of light intensity placed in 4 °C water bath. The decrease in OD_420_ afterduration of 1 min was immediately recorded with the spectrophotometer. A tube which wasplaced in complete darkness served as the reagent blank. The Hill activity was calculated as μmol of reduced K_3_Fe(SCN)_6_ h^−1^ mg^−1^chlorophyll. The content of the chlorophyll in the preparation was determined with the method described above.

### *In-vitro* analysis of osmolytes accumulation on photon availability at thylakoids (simulated experiment)

**Figure 15:**
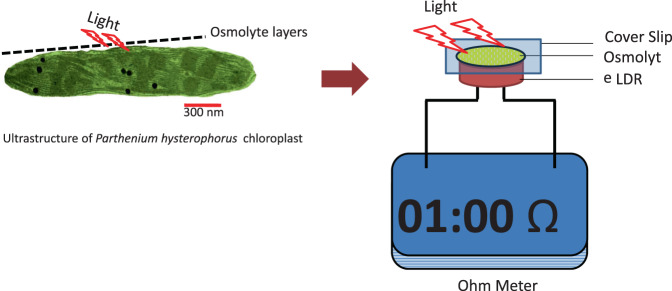
Impact of osmolytes accumulation on resistance (ohm) measured using a light-dependent resistor (LDR). The above model is proposed to exhibit the possible condition of the availability of photons in a cell/chloroplast with changing concentrations of osmolytes.

Combinations of two osmolytes viz. sucrose and proline in different concentrations (0.1, 0.2, 0.3, 0.5 and 1.0 M) were used in this experiment. Thin film of 100 µL of osmolytes was placed on the surface of photo cells of a light dependent resistor. A cover slip was placed to make a thin and uniform layer of the solution. The change in resistance of light dependent resistor was measured on the exposure of ordinary white light (250 µmol photons m^−2^ s^−1^) (Fig. 15). This experiment was conducted to demonstrate that osmolyte accumulation in the photosynthetic cells might affect the photosynthetic efficiency of the chloroplasts. Such suppression could be attributed to inhibited photon availability, if proven by this very original experiment.

### Fresh and dry weight estimations

For fresh weight and dry weight estimations, the entire plants were detached carefully from the soil, cleaned with DDW, followed blot-drying and weighed for fresh leaf weight per plant basis. For measuring dry matter accumulation, plants were kept in a hot-air oven at 65 °C for 3 days and dry weights were recorded. Dry weight was expressed as per cent change over control.

### Protein extraction from leaf

Total protein was extracted from the leaf tissue and processed as mentioned in Bagheri *et al*.^64^. Liquid nitrogen-frozen leaf was ground in a pre-chilled mortar and pestle to a fine powder and homogenized in extraction buffer (40 mM Tris-HCl pH 7.5, 2 mM of EDTA, 2% PVP, 0.07% β-mercaptoethanol, 1% Triton-X 100 and 1% PMSF). Homogenate was centrifuged at 13,148 × g at 4 °C for 20 min. Supernatant was treated with chilled 10% (w/v) TCA-acetone and left at 20 °C overnight followed by centrifugation at 6,708 × g for 10 min. The pellet was washed using chilled acetone.

A definite amount of protein was weighed and suspended in solubilization cocktail (40 mM Tris-Buffer,2 M thiourea, 7 mM urea, 40 mM DTT, 4% CHAPS and 2% Pharmalyte® with pH 3–10). A gentle stirring for around an hour at ambient temperature was performed and samples were centrifuged at 13,148 × g 4 °C for 10 min. Supernatant was immediately estimated for protein using Bradford reagent, before been loaded on the IPG strips.

### 1st and 2nd dimensional run of 2DE and Image analysis

Isoelectric focusing (IEF) was carried out as described by Schlesier and Mock^65^. Immobilized pH gradient (IPG) strips (ReadyStrip^TM^, Bio-Rad, USA) of 11 cm, non-linear with pH 4–7 were rehydrated passively over the night with 250 μg of protein sample. Isoelectric focusing of proteins was done by using a multi-step current flow to a completion of 70,000 Vh on IEF Cell™ (Bio-Rad, USA). Following the completion of IEF, equilibration of strips was done in DTT-buffer [50 mM of Tris-HCl (pH 8.8), 30% (v/v) of glycerol, 6 M of urea, 20 mM of DTT and 2% (w/v) of SDS] and then in IAA-buffer [50 mM of Tris-HCl (pH 8.8), 30% (v/v) of glycerin, 6 M of urea, 135 mM of Iodoacetamide and 2% (w/v) of SDS] for 15 mins each. Equilibrated IPG strips were loaded onto a 12% SDS polyacrylamide gel consisting of protein ladder (97,400 Da − 14,300 Da) at a constant current of 50 mA per gel and 20 °C using a protean II (Bio-Rad, USA) with upper (containing 1% SDS) and lower tank electrode buffers. As the electrophoretic run is completed, gels were stained in colloidal Blue Silver^66^.Coomassie Brilliant Blue (CBB) stain overnight. After staining and de-staining, the gels were digitized using Gel Documentation system (Bio-Rad, USA) for further analysis based on spot density and location. The images were analyzed using image master PDQuest software version 8.0, (Bio-Rad, USA). Only the spots showing significant quantitative changes more than 1.2 folds in abundance and with reproducible occurrence in three replicates were utilized for further analysis.

### In-gel tryptic digestion of proteins

Protein spots of our interest obtained from the 2D gel were picked for in-gel digestion by trypsin^67,68^. Following washing of these pieces with 100 µL of ultra-pure water, centrifuge was done at 151 × g for a span of 15 mins and washed again similarly. Then 50–100 µL of 50% (v/v) of acetonitrile was added and subjected to centrifugation at 151 × g for another 15 mins at 22–24 °C followed by repeating this step. To this, 5 µL of 1 M DTT and 49.5 µL of ammonium bicarbonate (20 mM) were added and kept for incubation for 45 mins at 56 °C in acetonitrile (ACN). Acetonitrile was discarded and to this 40 µL of IAA (55 mM) was added and centrifuged again at 151 × g/22–24 °C. Then 50% (v/v) of acetonitrile was added by disposing IAA and centrifuging at 151 × g and 24 °C. Acetonitrile was again discarded and 100% (v/v) acetonitrile was added followed by centrifugation at 151 × g for 15 min. The obtained pellet was vacuum dried. Incubation of samples was done in 20 µL of 1% (w/v) trypsin prepared in 20 mM of ammonium bicarbonate and incubated for an overnight at 37 °C. After an overnight protein digestion, centrifugation of samples was done at 151 × g for 15 mins. The supernatant was collected in fresh tube and to it an addition of 1% tetra-fluoroacetic acid (TFA) prepared in 50% (v/v) acetonitrile was done for mass spectrometric analysis.

### Peptide mass fingerprinting (PMF) and protein identification

Digested proteins in terms of peptides or peptide mass finger prints of differentially expressed proteins were collected and their analysis was done on ABI 4800 MALDI-TOF/TOF MS Analyzer (Applied Biosystems, USA), followed by identification (ID) of proteins using Result Dependent Analysis (RDA) of ABI GPS Explorer software, version 3.5 (Applied Biosystems, USA). Few important parameters set were as follows: MS (precursor-ion) peak filtering: 800–4000 m/z interval; Digestion enzyme: trypsin with one missed cleavage; monoisotopic, minimum signal-to-noise ratio (S/N) 10, mass tolerance 50 ppm. MS/MS (fragmentation) peak filtering: monoisotopic, MH^+^, minimum S/N, MS/MS fragment tolerance 0.2 Da; database used Viridiplantae (Green Plants) taxonomic subdatabase of ‘nr’ (nonredundant) database of the UniProtKB Swissprot database. During the primary stage of MS scan, scanning, analysis of data was performed as peptide mass fingerprinting (PMF), and preliminary protein ID was done through search against the database using the MASCOT (Matrix Science, http://www.matrixscience.com) algorithm. For evaluation of protein identification, proteins possessing significant scores were selected for result interpretation.

### Statistical analysis

In the present study, all the experiments were performed in replicates of five. The results were explained as mean ± standard error (SE). The significant difference at *P* ≤ 0.05 was determined by Tukey’s test. Hierarchical clustering, partial least squares discriminant analyses (PLS-DA) and principle component analyses were carried out using MetaboAnalyst 3.0 software. Mapping of protein network was made using the STRING system(http://string.embl.de), which is based upon confirmed and predicted interactions.

## Supplementary information

Supplementary Information.
