# Supplementary material for: Parthenium hysterophorus steps up Ca-regulatory pathway in defence against highlight intensities
Source: Sci Rep. 2020 Jun 2;10:8934. doi: 10.1038/s41598-020-65721-7 (PMC7265497; doi:10.1038/s41598-020-65721-7)
Supplement: Supplementary file 1 — Supplementary Information. [file 41598_2020_65721_MOESM1_ESM.docx]

***Parthenium hysterophorus* steps up Ca-regulatory pathway in defence against high light intensities**

Javed Ahmad^1^, M. Affan Baig^1^, Amna^1^, **Ibrahim A. Alaraidh^2^,** Abdulaziz A. Alsahli^2^ and M. Irfan Qureshi^1*^

^1^Department of Biotechnology, Jamia Millia Islamia, New Delhi-110 025, India

^2^Botany & Microbiology Department, Science College, King Saud University, P.O. Box 2455, Riyadh, Saudi Arabia

*Corresponding author: mirfanq@gmail.com

**
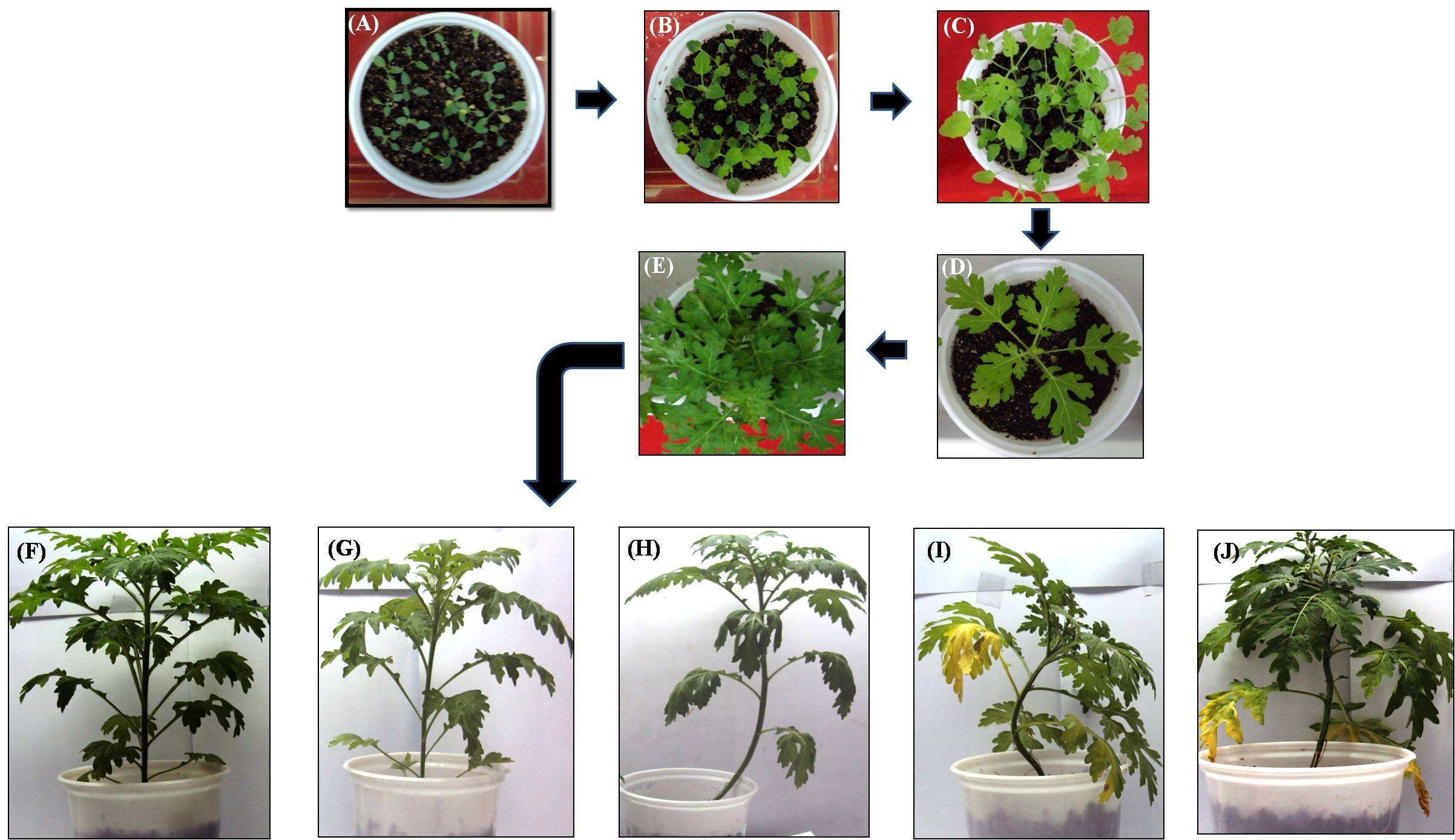
**

**Supplementary Figure S1.** Representative plants from different growth stages (A - E) and treated sets of *P*. *hysterophorus* (F- J): Set F. Control, Set G. three days after ML stress, Set H. five days after ML stress, Set I. three days after HL stress, Set J. five days after HL stress

**Functional annotation of differentially expressed proteins under ML stress**

Identified DEPs annotating with the help of Gene Ontology (GO) database and classified into biological process, molecular function and cellular component (Figure S2). Most of the up-regulated proteins were contributed as antioxidant molecular function (18%) whereas calcium dependent and chlorophyll binding proteins each of (20%) were more common as molecular function in down regulated proteome under ML stress. Stress responsive pathway (26%)/calcium signaling pathway (22%) was more enriched up-regulated GO term and signal transduction pathway was more enriched down-regulated GO term for biological processes under ML stress. Maximum sub-cellular localization of up-regulated proteins was contributed by chloroplast (20%), whereas maximum sub-cellular localization of down-regulated proteins was related to nucleus and plasma membrane (each of 22%) under ML stress.

**Functional annotation of differentially expressed proteins under HL stress**

Identified DEPs annotating with the help of Gene Ontology (GO) database and classified into biological process, molecular function and cellular component (Figure S3). Most of the up-regulated proteins were contributed as calcium dependent/binding molecular function (40%) whereas antioxidant proteins (16%) were more common as molecular function in down regulated proteins under HL stress. Calcium signaling pathway (40%) was more enriched up-regulated GO term and stress responsive pathway (21%) was more enriched down-regulated GO term for biological processes under HL stress. Maximum sub-cellular localization of up-regulated proteins was contributed by nucleus (43%), whereas maximum sub-cellular localization of down-regulated proteins was related to chloroplast (23%) under HL stress.


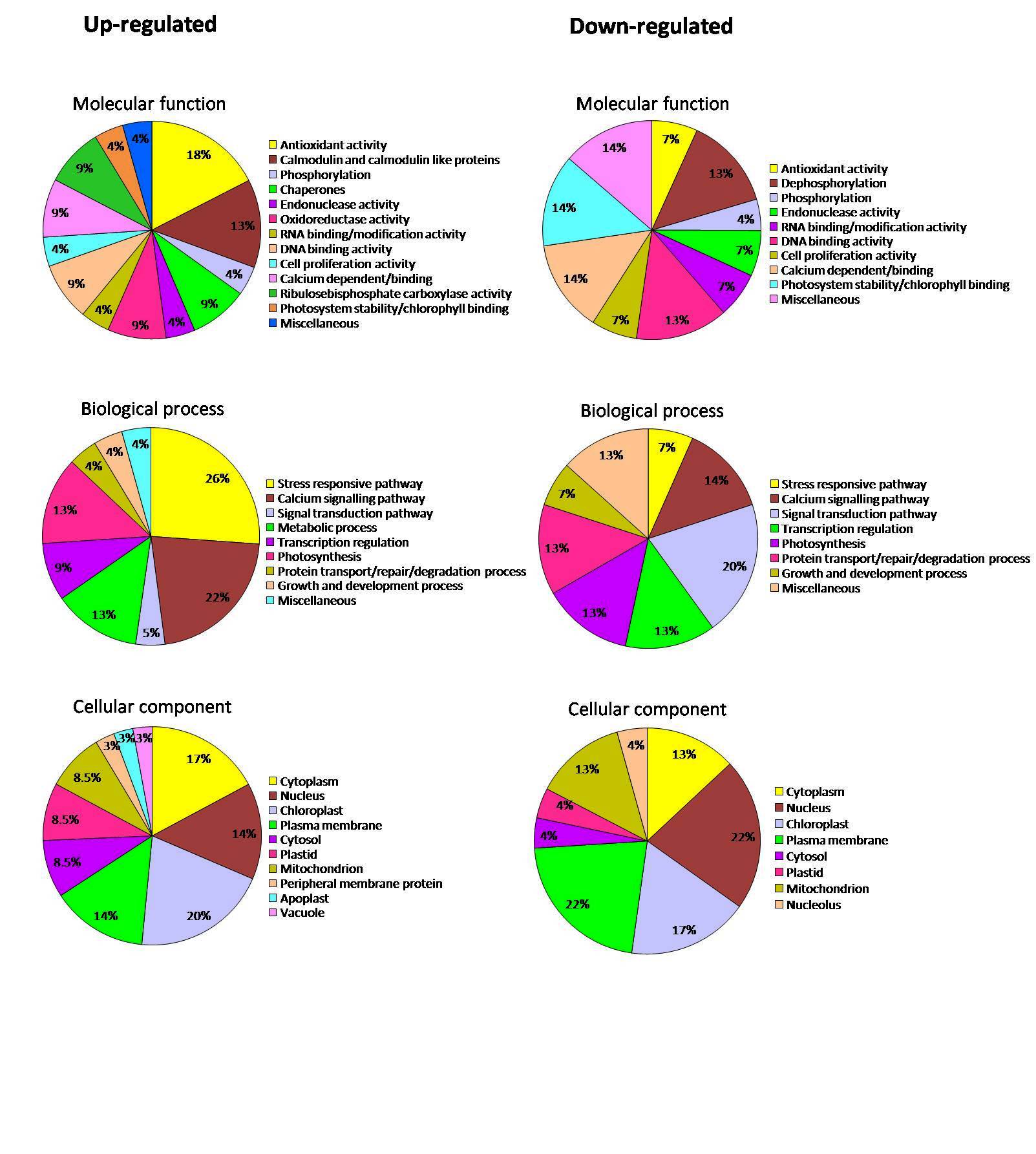


**Supplementary Figure S2.** Annotation of identified differentially expressed proteins classification by enrichment of Gene Ontology (GO) terms.


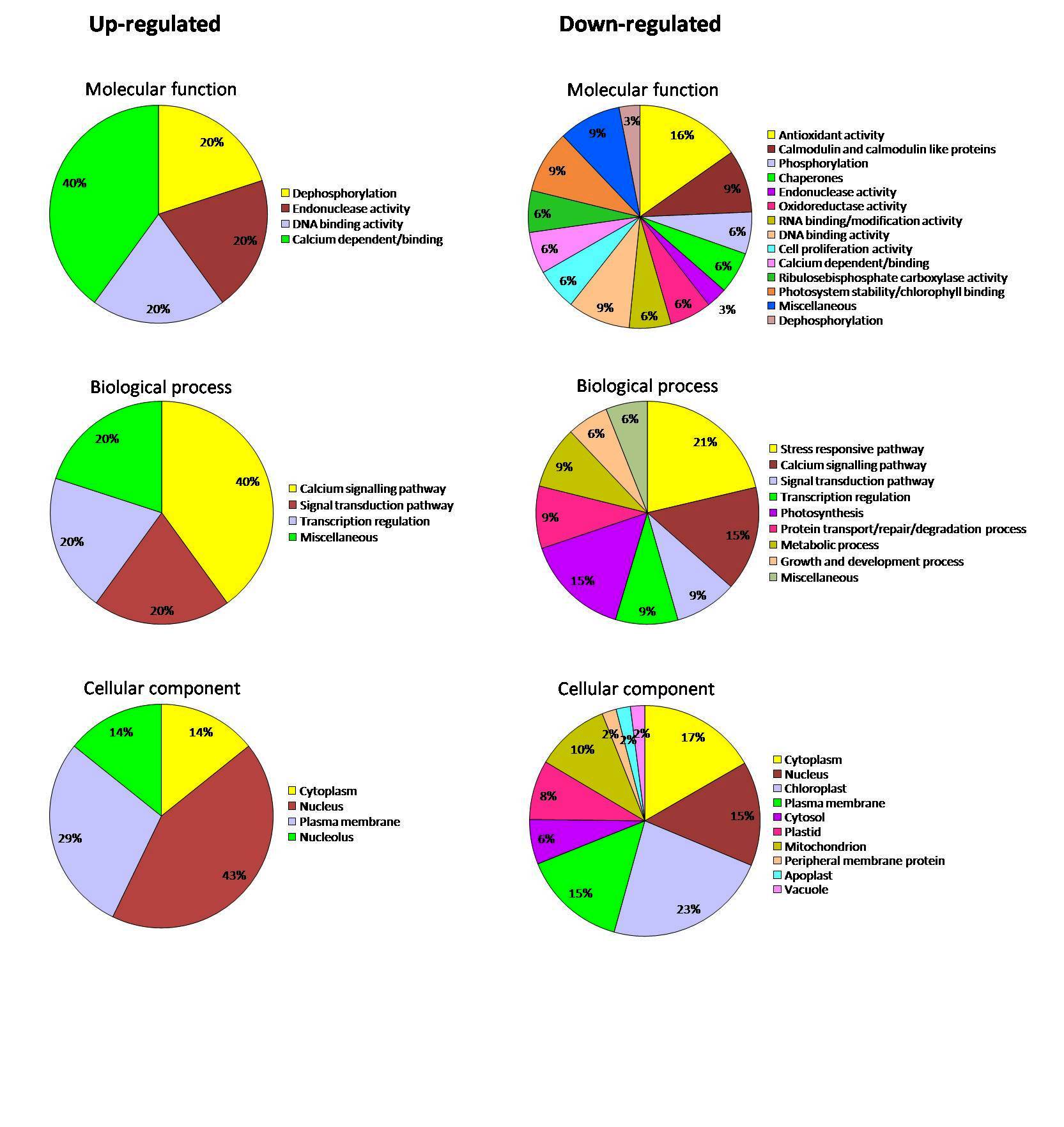


**Supplementary Figure S3.** Annotation of identified differentially expressed proteins classification by enrichment of Gene Ontology (GO) terms.


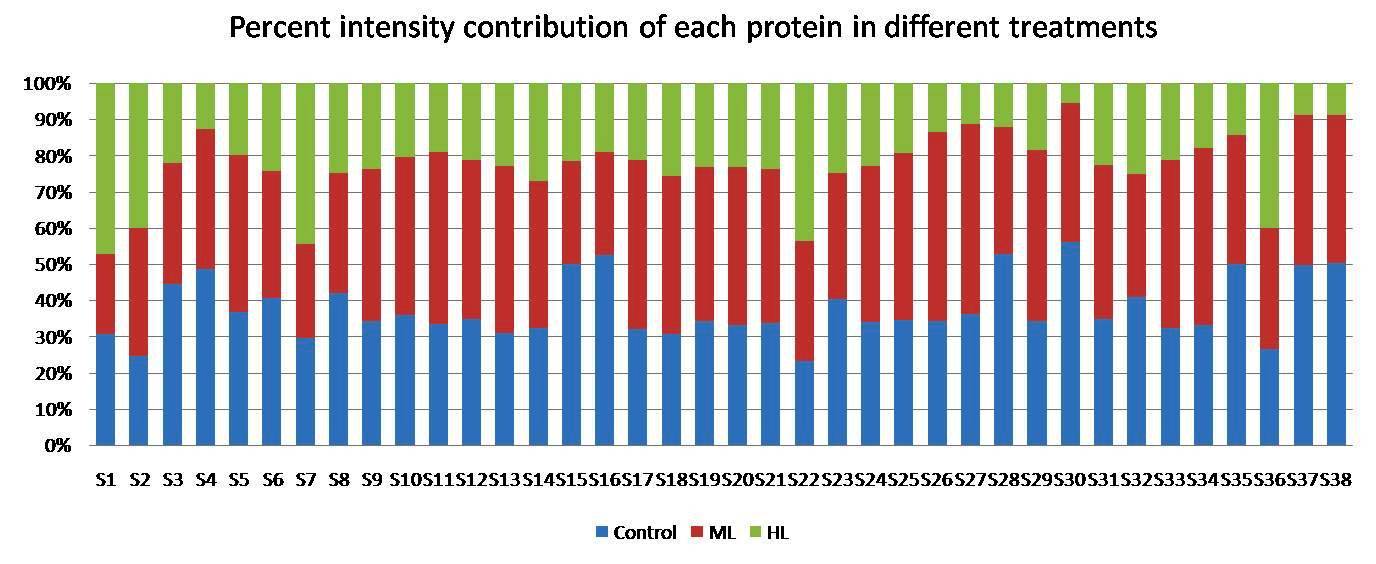


**Supplementary Figure S4.** Percent intensity contribution of each protein in different treatments. The intensities of differentially expressed proteins were obtained by PD-Quest analysis of 2D gels. Each bar represents intensity of a protein in control, ML and HL stressed leaf of *P. hysterophorus*.

**Table S1.** List of differentially expressed proteins identified in *P. hysterophorus* leaf under ML and HL stress. Spot IDs corresponds to the labeled 2D gels (Fig. 11).

| Spot I.D. | Protein name | Gene  name | Mascot  score | Location | Relative change  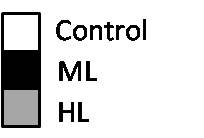 |
| --- | --- | --- | --- | --- | --- |
| S1 | Calcium dependent protein kinase 27 | ***CPK27*** | 78 | Plasma membrane | 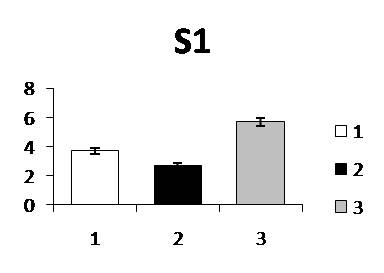 |
| S2 | Protein gamma response 1 | *GR1*  *F4F15.230* | 84 | Nucleus | 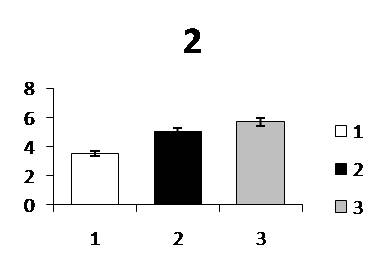 |
| S3 | Probable LRR receptor-like serine/threonine-protein kinase | *F2K11.19* | 73 | Plasma membrane | 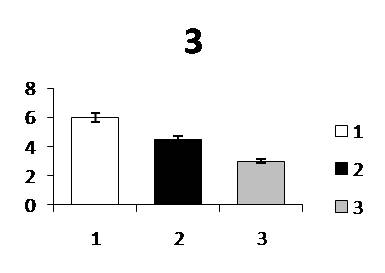 |
| S4 | Proton pump-interactor 2 | *PPI2*  *K7L4.14* | 59 | Plasma membrane | 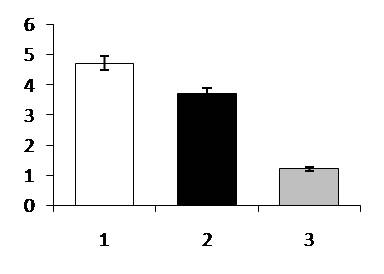 |
| S5 | Monodehydroascorbate reductase | *MDAR5*  *MDAR6* | 77 | Plastid  Chloroplast | 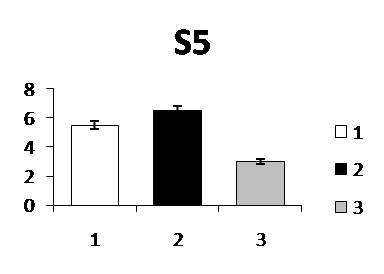 |
| S6 | Calcium-dependent protein kinase 6 | *CPK6* | 82 | Plasma membrane  Nucleus | 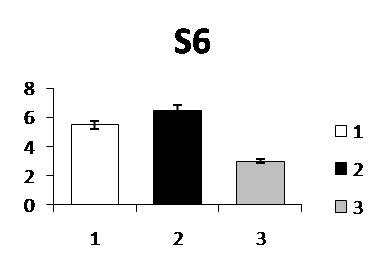 |
| S7 | Serine/threonine-protein phosphatase PP1 isozyme 3 | *TOPP3*  *F22C12.20* | 56 | Nucleus  Cytoplasm  Nucleolus | 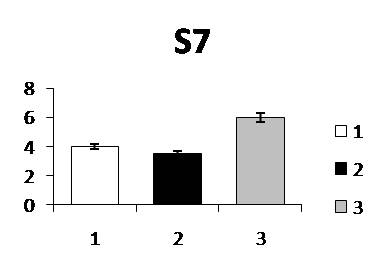 |
| S8 | Probable SAL3 phosphatase | *SAL3*  *MBM17.9* | 27 | Mitochondrion | 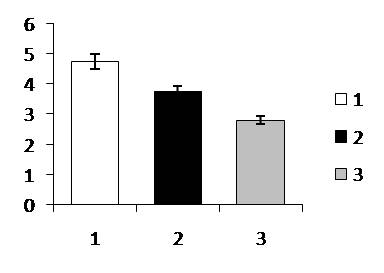 |
| S9 | Glutathione S-transferase U4 | *GSTU4*  *F16P2.16* | 66 | Cytoplasm | 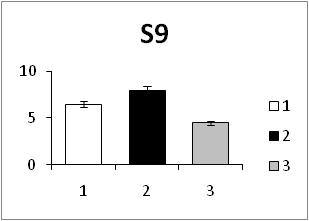 |
| S10 | Protein brevis radix like -1 | *BRXLI* | 55 | Plasma membrane  Nucleus  Periplasmic membrane | 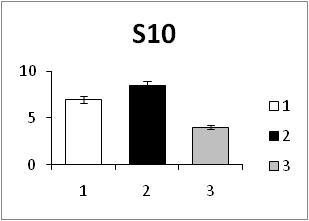 |
| S11 | Large subunit of ribulose-1,5-bisphosphate carboxylase/oxygenase | *rbcL* | 93 | Chloroplast | 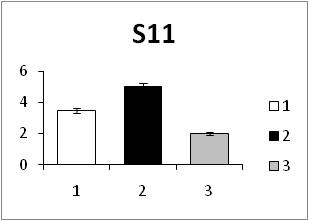 |
| S12 | RNA pseudouridine synthase 1 | *F13N6.19*  *F14G9.4* | 43 | Mitochondrion | 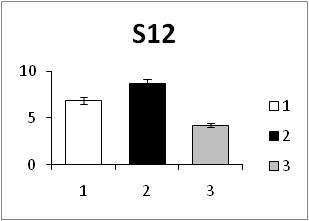 |
| S13 | Calmodulin-like protein 1 | *CML1*  *Os01g0810300* | 68 | Plasma membrane | 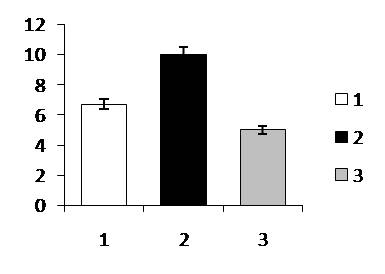 |
| S14 | Glutathione S-transferase 1 | *GSTA1* | 86 | Cytoplasm | 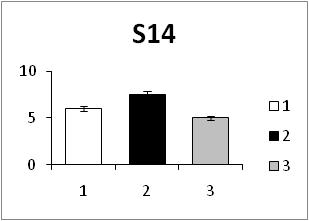 |
| S15 | Auxin-responsive protein IAA7 | *IAA7*  *LOC_Os02g13520* | 54 | Nucleus | 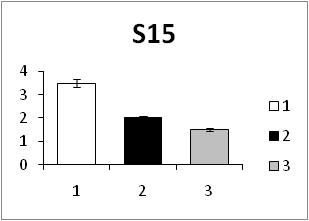 |
| S16 | Basic leucine zipper 6 | *BZIP06*  *LOC_Os01g55150* | 28 | Nucleus | 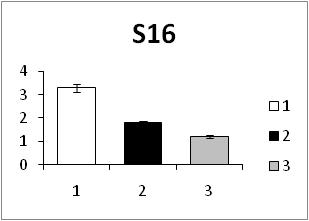 |
| S17 | Probable WRKY transcription factor 74 | *WRKY74*  *F414.30* | 56 | Nucleus | 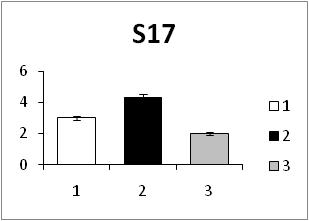 |
| S18 | Shikimate kinase 1 | *SK1*  *F7D8.26* | 28 | Plastid  Chloroplast | 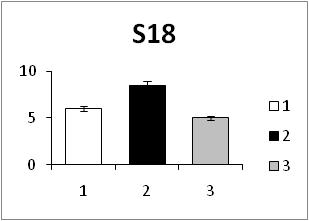 |
| S19 | ATP-dependent Clp protease proteolytic subunit | *CIpP* | 32 | Plastid  Chloroplast | 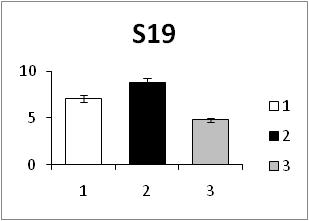 |
| S20 | 17.6 kDa class I heat shock protein | *Hsp17.6* | 55 | Cytoplasm | 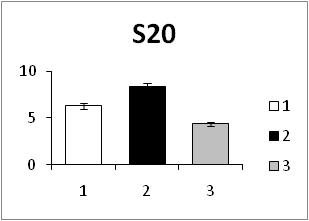 |
| S21 | Photosystem I assembly protein ycf4 | ***Ycf4*** | 46 | Chloroplast | 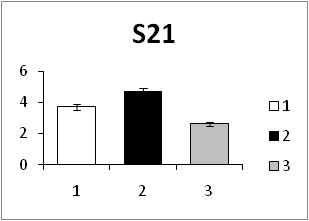 |
| S22 | Probable calcium-binding protein CML15 | *CML15*  *LOC_Os05g31620* | 88 | Plasma membrane | 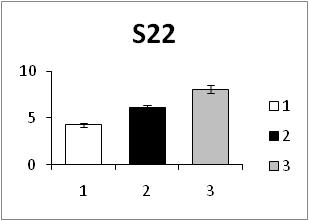 |
| S23 | Acyl carrier protein 2 | *MTACP2*  *T8F5.6* | 26 | Mitochondrion | 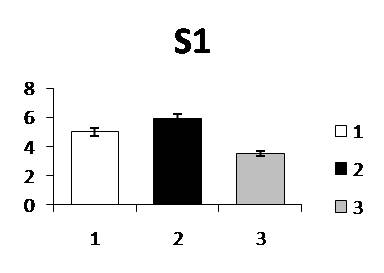 |
| S24 | Desiccation-related protein | - | 62 | Cytosol  Plasma membrane  Apoplast | 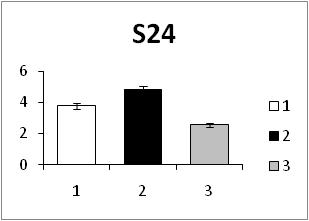 |
| S25 | Calmodulin-like protein 7 | *CML7*  *T21E18.4* | 75 | Nuclear membrane  Vacuole  Cytosol | 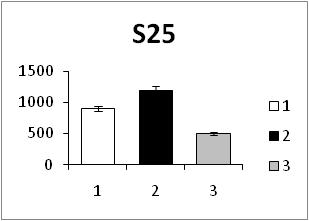 |
| S26 | Thioredoxin H4-1 | *Os01g0168200*  *LOC_Os01g07376* | 84 | Cytoplasm | 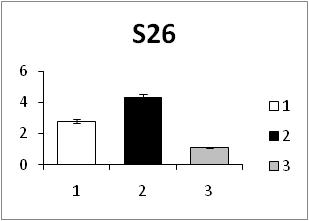 |
| S27 | **Calmodulin-1** | *CALM1* | 93 | Plasma membrane  Cytoplasm | 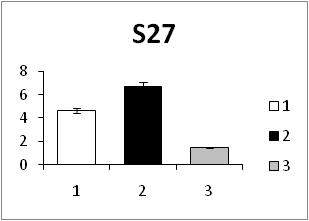 |
| S28 | Oxygen-evolving enhancer protein 2 | *PSBP1* | 61 | Chloroplast | 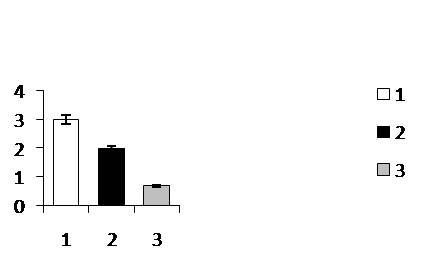 |
| S29 | Succinate-semialdehyde dehydrogenase | *ALDH5F1*  *SSADH1*  *T8K14.14* | 23 | Chloroplast  Mitochondrion | 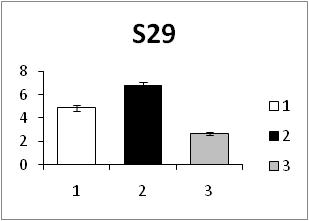 |
| S30 | Peptide methionine sulfoxide reductase B4 | *MSRB4* | 46 | Cytoplasm  cytososol | 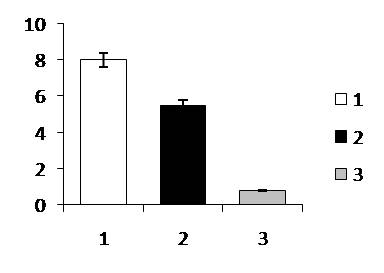 |
| S31 | Polyamine oxidase 1 | *PAO1, PAO*  *MSH12.17* | 27 | Mitochondrion | 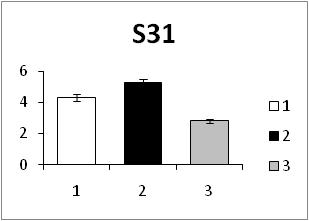 |
| S32 | Pentatricopeptide repeat-containing protein | *PCMP-H40* | 31 | Chloroplast  Mitochondrion | 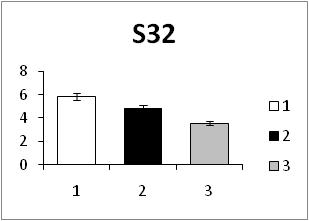 |
| S33 | Probable calcium-binding protein CML28 | ***CML28*** | 86 | Cytoplasm | 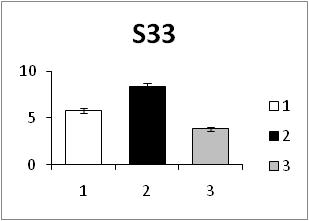 |
| S34 | Small subunit of ribulose-1,5-bisphosphate carboxylase/oxygenase | ***RBCS*** | 34 | Chloroplast | 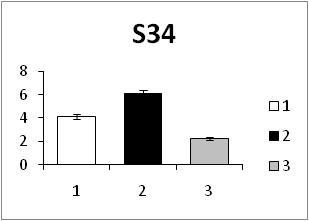 |
| S35 | Photosystem II protein J | ***psbJ*** | 27 | Chloroplast | 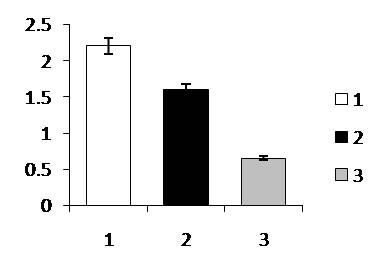 |
| S36 | Cyclic dof factor 4 | *CDF4*  *DOF2.3* | 25 | Nucleus | 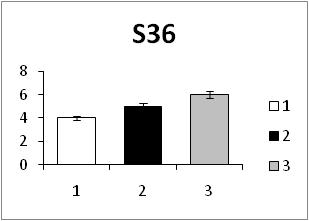 |
| S37 | Lactoylglutathione lyase | *GLY1* | 64 | Plastid  Chloroplast | 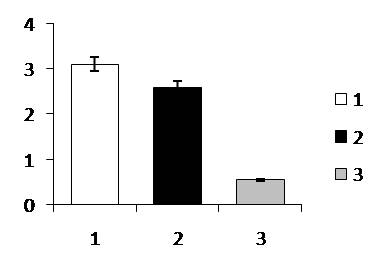 |
| S38 | WPP domain containing protein 3 | *WPP3* | 48 | Cytoplasm  Nucleus | 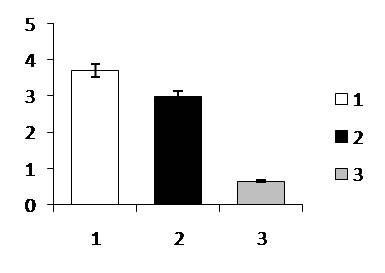 |

**Supplementary Figure S5.** 2-DE uncroped gel images (cropped gel images of same are used in main article file) of control versus moderate (ML; 500 µmol photons m^-2^ s^-1^) and high (HL; 1000 µmol photons m^-2^ s^-1^) light intensities exposed *Parthenium hysterophorus* at five days after treatments (5 DAT).
